# Association between inflammatory markers and bone mineral density: a cross-sectional study from NHANES 2007–2010

**DOI:** 10.1186/s13018-023-03795-5

**Published:** 2023-04-17

**Authors:** Shuai Chen, Xiaohe Sun, Jie Jin, Guowei Zhou, Zhiwei Li

**Affiliations:** 1 Department of Orthopaedics, The Second Affiliated Hospital of Nanjing University of Chinese Medicine, No.23, Nanhu Road, Jianye District, Nanjing, 210017 Jiangsu Province People’s Republic of China; 2 Department of Oncology, Jiangsu Province Hospital of Chinese Medicine, Affiliated Hospital of Nanjing University of Chinese Medicine, Nanjing, People’s Republic of China; 3 Department of General Surgery, Jiangsu Province Hospital of Chinese Medicine, Affiliated Hospital of Nanjing University of Chinese Medicine, No.155, Hanzhong Road, Qinhuai District, Nanjing, 210029 Jiangsu Province People’s Republic of China

**Keywords:** Osteoporosis, Bone mineral density, Inflammatory markers, Platelet-to-lymphocyte ratio, NHANES

## Abstract

**Purpose:**

Monocyte-to-lymphocyte ratio (MLR), neutrophil-to-lymphocyte ratio (NLR), platelet-to-lymphocyte ratio (PLR) are acknowledged as novel inflammatory markers. However, studies investigating the correlation between inflammatory markers and osteoporosis (OP) remain scarce. We aimed to investigate the relationship between NLR, MLR, PLR and bone mineral density (BMD).

**Methods:**

A total of 9054 participants from the National Health and Nutrition Examination Survey were included in the study. MLR, NLR and PLR were calculated for each patient based on routine blood tests. Given the complex study design and sample weights, the relationship between inflammatory markers and BMD was evaluated through weighted multivariable-adjusted logistic regression and smooth curve fittings. In addition, several subgroup analyses were conducted to assess the robustness of the outcomes.

**Results:**

This study observed no significant relationship between MLR and lumbar spine BMD (*P* = 0.604). However, NLR was positively correlated with lumbar spine BMD (β = 0.004, 95% CI: 0.001 to 0.006, *P* = 0.001) and PLR was negatively linked to lumbar spine BMD (β = − 0.001, 95% CI: − 0.001 to − 0.000, *P* = 0.002) after accounting for covariates. When bone density measurements were changed to the total femur and femoral neck, PLR was still significantly positively correlated with total femur (β = − 0.001, 95% CI: − 0.001, − 0.000, *P* = 0.001) and femoral neck BMD (β = − 0.001, 95% CI: − 0.002, − 0.001, *P* < 0.001). After converting PLR to a categorical variable (quartiles), participants in the highest PLR quartile had a 0.011/cm^2^ lower BMD than those in the lowest PLR quartile (β = − 0.011, 95% CI: − 0.019, − 0.004,* P* = 0.005). According to subgroup analyses stratified by gender and age, the negative correlation with PLR and lumbar spine BMD remained in males and age < 18 groups, but not in female and other age groups.

**Conclusions:**

NLR and PLR were positively and negatively correlated with lumbar BMD, respectively. And PLR might serve as a potential inflammatory predictor of osteoporosis outperforming MLR and NLR. The complex correlation between the inflammation markers and bone metabolism requires further evaluation in large prospective studies.

**Supplementary Information:**

The online version contains supplementary material available at 10.1186/s13018-023-03795-5.

## Introduction

Osteoporosis is a systemic bone metabolic disease characterized by diminished bone mineral density (BMD) and bone microstructure destruction [1, 2]. As the population ages, the incidence of osteoporosis is increasing year by year worldwide, and is ranked seventh in terms of incidence, bringing a huge impact on the lives of the elderly [3]. According to the International Osteoporosis Foundation, approximately 200 million individuals worldwide are affected by osteoporosis. Furthermore, it is expected that the prevalence of osteoporosis will increase by 310% in men and 240% in women by 2050 [4]. It is estimated that up to 46% have low bone density and up to 11% have osteoporosis at either the femoral neck or spine in Americans aged 50 and older [5]. Research indicates osteoporosis-related medical expenditures in the global medical expenditures are on the rise [6]. In addition, global spending on osteoporotic fractures will increase from $10 billion in 2010 to $17 billion in 2030 every year [7, 8]. Thus, applying an objective, accurate, affordable, and convenient method to predict the occurrence of early osteoporosis is of paramount importance.

Bone immunology has demonstrated that inflammation plays a critical role in the pathogenesis of osteoporosis and fragility fractures [9], which might be related to age-related oxidative stress and low activation of the immune system. Inflammation of dysfunctional lymphocytes could initiate a cascade reaction of inflammatory cytokines and chemokines, leading to the aggregation of neutrophils and macrophages, thus breaking the dynamic balance of bone formation [10–12]. Besides, recent studies have shown that inflammatory cytokines act on mesenchymal stem cells and osteoclast precursors to enhance osteoclast mediated bone resorption, such as C-reactive protein (CRP), Interleukin-1 (IL-1), and Tumor Necrosis Factor-α(TNF-α) [13]. Therefore, the monitoring of chronic inflammation might be crucial for both the early prediction and diagnosis of osteoporosis.

Monocyte-to-lymphocyte ratio (MLR), neutrophil-to-lymphocyte ratio (NLR), and platelet-to-lymphocyte ratio (PLR) are newly proposed inflammatory factors based on the full blood count (FBC), which can better reflect the body's immune and inflammatory status [14]. Given that blood routine examination is easily available and low cost, NLR, PLR, and MLR have become simple, inexpensive, and reliable markers of inflammation [15]. And these inflammatory markers have been proven to play pivotal roles in the risk and prognosis assessment of ankylosing spondylitis, cardiovascular diseases, and malignant tumors [16–18]. In addition, a cross-sectional study of 648 older adults showed that high levels of NLR and PLR were closely correlated with the prevalence of osteoporosis, suggesting that NLR and PLR are potential targets for osteoporosis screening [19]. Similarly, Tang et al. reported that systemic immune-inflammation index (SII) and PLR serve as valuable and convenient inflammatory markers that can be used to predict the risk of low BMD or osteoporosis in postmenopausal women [20]. However, Lee et al. demonstrated that NLR was adversely related to lumbar spine BMD, but no significant relationship between PLR and BMD was observed [21]. Therefore, in order to provide more evidence to resolve the above contradictions, we investigated the correlation between NLR, MLR, PLR and BMD based on data from the National Health and Nutrition Examination Survey (NHANES) from 2007 to 2010.

## Methods

### Study population

The NHANES was conducted by the Centers for Disease Control and Prevention (CDC) that consists of a series of independent, nationally representative cross-sectional surveys designed to assess the health and nutritional status of Americans (http://www.cdc.gov/nchs/nhanes/) [22]. All participants signed the consent form and provided demographic information, examination data, laboratory tests and questionnaire data. For our study, data were selected in two cycles of the NHANES survey (2007–2008, 2009–2010). In addition, all NHANES protocols were approved by the National Center for Health Statistics (NCHS) Research Ethics Review Board and informed consent was obtained from all participants [23].

**Fig. 1:**
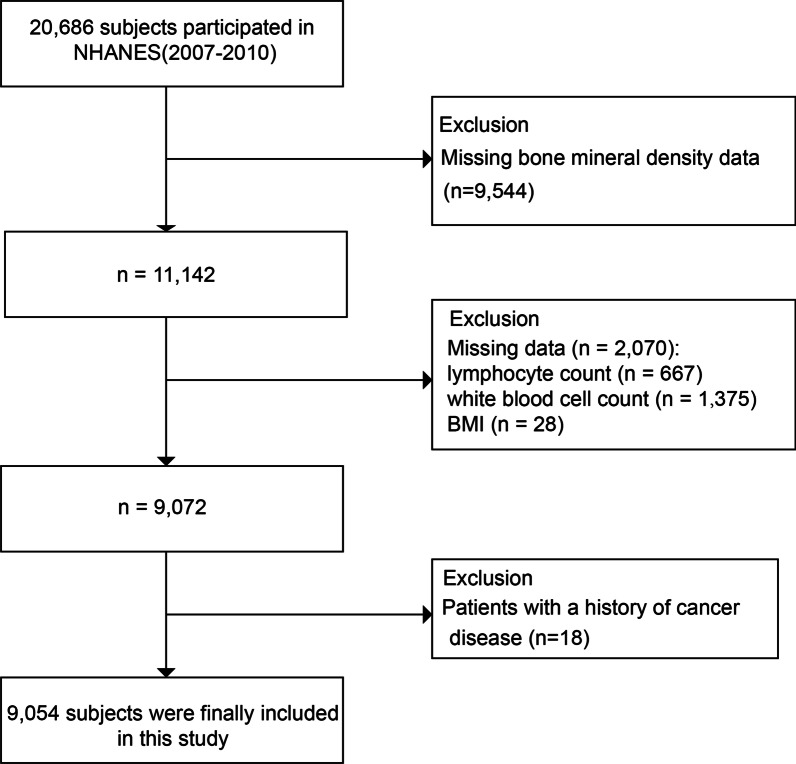
Study flowchart. NHANES, National Health and Nutrition Examination Survey

Study participants who completed demographic information, health questionnaires, laboratory tests, and BMD measurements (n = 20,686) were included. The exclusion criteria were as follows (Fig. 1): (1) we excluded 9544 people without data on BMD; (2) we excluded 2070 participants who had missing data on lymphocyte count, white blood cell count, and BMI; (3) we excluded 18 participants with a previous cancer history. Ultimately, a total of 9054 individuals were recruited.

### BMD examination

As the outcome variables of this study, total femur, femoral neck, and lumbar spine BMD was measured by dual-energy X-ray absorption (DXA) with a Hologic QDR 4500A fan beam densitometer (Hologic Inc., Bedford, MA, USA) [24]. All measurements were taken by radiologic technologists who are certified and trained to administer DXA examinations. In the survey, all participants aged 8 and older were eligible to receive a DXA scan. DXA is not suitable for pregnant women, individuals weighing over 300 pounds, or those who have taken radiographic contrast material in the past 7 days.

### Serum measurements

The morning fasting venous blood of all participants was collected for routine clinical chemistry analysis. Lymphocyte, monocyte, and platelet count in 1000 cells/μL were obtained from the whole blood using the Coulter counter method. MLR = monocyte count/lymphocyte count; NLR = neutrophil count/lymphocyte count; PLR = platelet count/lymphocyte count [25].

### Covariates

Age, gender, race/ethnicity, and education level of participants were collected with questionnaires in survey interviews. The mean age was 39.54 years ranging from 12 to 80 years. Race was classified as Mexican American, non-Hispanic white, non-Hispanic black, or another race. Education level was categorized as less than high school, high school, more than high school or missing. Additionally, laboratory data included glycosylated hemoglobin, Type A1C (HbA1c, %), albumin (ALB, g/dL), blood urea nitrogen (BUN, mg/dL), total cholesterol (TC, mg/dL), triglycerides (TG, mg/dL), total calcium (Ca mg/dL), creatinine (Cr, mg/dL), phosphorus (P, mg/dL), uric acid (UA, mg/dL), segmented neutrophils number (1000 cells/uL), red blood cell count (million cells/uL), hemoglobin (Hb, g/dL), and platelet count (PLT, 1000 cells/uL). Finally, questionnaire data covered alcohol consumption, smoking status, and BMI (kg/m^2^). Alcohol consumption was divided into non-drinkers, low to moderate drinkers, and heavy drinkers. Smoking status was classified as current, never or former.

### Statistical analyses

When it came to statistical analysis, the complex survey design elements of the NHANES, including weighting, clustering, and stratification, were taken into consideration in accordance with the NCHS normative analysis guidelines. To begin with, participants were grouped according to the quartiles of lumbar-spine BMD level and we conducted the linear regression model and the weighted chi-square test to test the statistical differences between groups of lumbar-spine BMD levels with all the variables. In the next step, we incorporated statistically significant covariates into the multiple linear regression model. The association between inflammatory markers and BMD was further analyzed, and the effect value β and its 95% confidence interval (CI) were calculated by accounting for potential confounding factors such as demographic information, laboratory tests and examination data. Model 1 had no adjustments. Model 2 was adjusted for gender, age, and race. Model 3 was further adjusted for education, smoking behavior, alcohol consumption, BMI, HbA1c, ALB, BUN, TC, TG, Ca, Cr, P, and UA. Moreover, PLR was converted into categorical variables based on quartiles, and then calculated the *P* for trend by taking the mean of PLR quartiles as the continuous variable. Additionally, through smoothing curve fitting and generalized additive models, the linear relationship between NLR, PLR, and lumbar spine BMD was further examined. Due to the association between PLR and lumbar spine BMD remained significant after adjustment for different covariates. Finally, we performed subgroup analyses to examine the robustness of the correlation between PLR and lumbar spine BMD. For missing values in covariates, median interpolation was used for continuous variables, and third categories were added for classified variables to assess the effect of missingness on the outcome. In the study, Empower Stats and R software were used for data analysis. The detailed code for the data analysis was presented in Additional file 1.

## Results

### The baseline characteristics of the participants

As presented in Table 1, we presented the sociodemographic and clinical characteristics of 9054 participants in four groups of lumbar spine BMD. Male, Mexican American, well-educated, non-drinkers, and non-smokers were more likely to be in the upper quartile of BMD. In addition, people with elevated BMD levels tended to have higher levels of BMI, HbA1c, TG, Cr, UA, segmented neutrophils number, red blood cell count and NLR. In contrast, they had lower levels of Ca, P, and PLR.

**Table 1:** Baseline characteristics of the research population based on lumbar spine BMD quartiles

	Quartiles of lumbar-spine BMD				*P* value
	Q1 (≤ 0.919)	Q2 (0.919–1.019)	Q3 (1.019–1.115)	Q4 (≥ 1.15)	
*Demographics*					
Age (years)	41.16 ± 20.99	38.96 ± 17.06	37.30 ± 15.79	40.17 ± 15.43	< 0.001
*Gender*					
Male	1041 (46.42%)	1093 (48.06%)	1150 (51.03%)	1226 (53.67%)	< 0.001
Female	1202 (53.58%)	1181 (51.94%)	1103 (48.97%)	1058 (46.33%)	
Race/ethnicity (%)					< 0.001
Non-hispanic white	279 (12.42%)	266 (11.70%)	212 (9.41%)	155 (6.80%)	
Non-hispanic black	364 (16.23%)	314 (13.79%)	230 (10.20%)	220 (9.63%)	
Mexican American	1465 (65.33%)	1517 (66.72%)	1562 (69.34%)	1524 (66.73%)	
Another race	135 (6.02%)	177 (7.79%)	249 (11.05%)	385 (16.84%)	
Level of education (%)					< 0.001
Less than high school Grade	339 (15.13%)	410 (18.04%)	339 (15.04%)	310 (13.58%)	
High school grade	410 (18.27%)	416 (18.30%)	409 (18.16%)	509 (22.30%)	
More than high school Grade	891 (39.19%)	1082 (47.56%)	1185 (52.61%)	1276 (55.86%)	
Missing	593 (26.41%)	366 (16.10%)	320 (14.19%)	189 (8.26%)	
*Examination and questionnaire data*					
Alcohol consumption (%)					< 0.001
Nondrinker	1108 (49.37%)	1381 (60.72%)	1499 (66.53%)	1634 (71.55%)	
Low to moderate drinker	462 (20.61%)	417 (18.36%)	375 (16.65%)	364 (15.94%)	
Heavy drinker	673 (30.01%)	476 (20.92%)	379 (16.82%)	286 (12.51%)	
Smoking status (%)					< 0.001
Current	762 (33.94%)	852 (37.45%)	841 (37.31%)	942 (41.26%)	
Never	897 (40.01%)	1057 (46.50%)	1091 (48.43%)	1153 (50.47%)	
Former	584 (26.05%)	365 (16.05%)	321 (14.25%)	189 (8.27%)	
BMI (kg/m^2^)	24.50 ± 5.28	26.34 ± 5.34	27.65 ± 5.61	29.19 ± 5.54	< 0.001
*Laboratory indices*					
HbA1c (%)	5.49 ± 0.70	5.44 ± 0.73	5.45 ± 0.77	5.53 ± 0.84	< 0.001
Albumin (g/dL)	4.35 ± 0.30	4.35 ± 0.30	4.32 ± 0.32	4.29 ± 0.32	< 0.001
Blood urea nitrogen (mg/dL)	12.40 ± 4.74	12.26 ± 4.33	12.11 ± 4.31	12.44 ± 4.71	0.049
Total cholesterol (mg/dL)	191.32 ± 45.17	192.15 ± 40.66	189.81 ± 40.15	190.53 ± 38.90	0.243
Triglycerides (mg/dL)	135.83 ± 109.39	141.39 ± 118.63	139.73 ± 107.48	153.65 ± 132.44	< 0.001
Total calcium (mg/dL)	9.52 ± 0.36	9.49 ± 0.36	9.50 ± 0.36	9.41 ± 0.36	< 0.001
Creatinine (mg/dL)	0.79 ± 0.32	0.84 ± 0.26	0.86 ± 0.33	0.89 ± 0.31	< 0.001
Uric acid (mg/dL)	5.05 ± 1.25	5.34 ± 1.36	5.37 ± 1.35	5.54 ± 1.37	< 0.001
Phosphorus (mg/dL)	4.07 ± 0.69	3.83 ± 0.60	3.81 ± 0.59	3.73 ± 0.56	< 0.001
Lymphocyte number (1000 cells/uL)	2.13 ± 0.67	2.15 ± 0.65	2.15 ± 0.65	2.18 ± 1.11	0.306
Monocyte number (1000 cells/uL)	0.54 ± 0.17	0.55 ± 0.19	0.55 ± 0.18	0.55 ± 0.18	0.041
Segmented neutrophils number (1000 cells/uL)	3.90 ± 1.55	4.14 ± 1.58	4.21 ± 1.76	4.27 ± 1.81	< 0.001
Red blood cell count (million cells/uL)	4.66 ± 0.45	4.71 ± 0.47	4.72 ± 0.49	4.72 ± 0.46	< 0.001
Hb (g/dL)	14.22 ± 1.32	14.41 ± 1.44	14.37 ± 1.50	14.36 ± 1.49	< 0.001
PLT count (1000 cells/uL)	260.48 ± 69.65	254.65 ± 63.66	254.96 ± 62.24	254.02 ± 63.27	0.004
*Inflammatory markers*					
MLR	0.27 ± 0.10	0.27 ± 0.11	0.27 ± 0.11	0.27 ± 0.11	0.604
NLR	1.97 ± 1.00	2.06 ± 0.98	2.11 ± 1.13	2.12 ± 1.02	< 0.001
PLR	132.36 ± 52.53	127.24 ± 46.86	128.37 ± 47.16	127.11 ± 47.22	< 0.001

Mean ± SD for continuous variables: the *P* value was calculated by the weighted linear regression model. (%) for categorical variables: the *P* value was calculated by the weighted chi-square test

*BMD* bone density mineral; *BMI* body mass index; *Hb* hemoglobin; *PLT* platelet; *MLR* monocyte-to-lymphocyte ratio; *NLR* neutrophil-to-lymphocyte ratio; *PLR* platelet-to-lymphocyte ratio

### Associations between inflammatory markers and BMD in each part of the body

As shown in Table 2, we showed the adjusted correlation between NLR, PLR and BMD in each part of the body. In model 3, NLR was positively correlated with lumbar spine BMD (β = 0.004, 95% CI: 0.001 to 0.006, *P* = 0.001) and PLR was negatively linked to lumbar spine BMD (β = − 0.001, 95% CI: − 0.001 to − 0.000, *P* = 0.002). Furthermore, we observed that PLR was significantly associated with BMD at the lumbar spine, total femur, and femur neck (*P* < 0.005). However, if the BMD measurement site is changed to the total femur and femoral neck, no significant relationship was found between NLR and BMD. In addition, we treated PLR as categorical variables (quartiles) for sensitivity analysis (Table 3). We found that significant associations between PLR and lumbar BMD across all the quartile categories and the level of BMD increased with the decreasing extent of PLR. Participants in the Q4 group had 0.011 g/cm^2^ lower lumbar BMD than those in the Q1 group in model 3. And the general trends were consistent among different quartile groups (*P for trend* < 0.01).

**Table 2:** Associations between PLR, NLR and BMD in each part of the body

	Lumbar-spine BMD	Total femur BMD	Femoral neck BMD
*NLR*			
Model 1 β (95% CI)	0.007 (0.004, 0.010) < 0.001	− 0.003 (− 0.007, − 0.000) 0.028	− 0.0023 (− 0.006, 0.001) 0.139
Model 2 β (95% CI)	0.009 (0.006, 0.012) < 0.001	0.002 (− 0.001, 0.005) 0.122	0.003 (− 0.001, 0.006) 0.127
Model 3 β (95% CI)	0.004 (0.001, 0.006) 0.001	− 0.002 (− 0.005, 0.000) 0.100	− 0.003 (− 0.006, 0.000) 0.060
*PLR*			
Model 1 β (95% CI)	− 0.001 (− 0.002, − 0.001) < 0.001	− 0.003 (− 0.004, − 0.003) < 0.001	− 0.004 (− 0.005, − 0.003) < 0.001
Model 2 β (95% CI)	− 0.001 (− 0.002, − 0.001) < 0.001	− 0.002 (− 0.002, − 0.001) < 0.001	− 0.002 (− 0.003, − 0.001) < 0.001
Model 3 β (95% CI)	− 0.001 (− 0.001, − 0.000) 0.002	− 0.001 (− 0.001, − 0.000) 0.001	− 0.001 (− 0.002, − 0.001) < 0.001

Logistic regression models: Model 1: no covariates were adjusted. Model 2 was adjusted for demographic factors, including gender, age, race. Model 3 was adjusted for gender, age, race, education, smoking behavior, alcohol consumption, BMI, HbA1c, albumin, blood urea nitrogen, total cholesterol, triglycerides, total calcium, creatinine, phosphorus, uric acid

**Table 3:** Subgroup analyses stratified by gender and race

	Model 1 β (95% CI)	Model 2 β (95% CI)	Model 3 β (95% CI)
*PLR (quartile)*			
Q1	Reference	Reference	Reference
Q2	− 0.005 (− 0.014, 0.004) 0.239	− 0.004 (− 0.013, 0.004) 0.336	− 0.002 (− 0.010, 0.006) 0.642
Q3	− 0.010 (− 0.019, − 0.002) 0.018	− 0.009 (− 0.017, − 0.001) 0.038	− 0.006 (− 0.014, 0.001) 0.108
Q4	− 0.014 (− 0.023, − 0.005) 0.001	− 0.014 (− 0.023, − 0.006) 0.001	− 0.011 (− 0.019, − 0.004) 0.005
*P* for trend	< 0.001	< 0.001	< 0.001
*Subgroup analysis stratified by gender*			
Male	− 0.002 (− 0.003, − 0.001) < 0.001	− 0.003 (− 0.004, − 0.002) < 0.001	− 0.002 (− 0.003, − 0.001) < 0.001
Female	− 0.000 (− 0.001, 0.000) 0.395	0.000 (− 0.001, 0.001) 0.822	0.000 (− 0.001, 0.001) 0.596
*Subgroup analysis stratified by age*			
Age < 18	− 0.003 (− 0.004, − 0.001) 0.006	− 0.002 (− 0.004, − 0.001) 0.004	− 0.001 (− 0.001, − 0.000) 0.002
Age 18–45	0.000 (− 0.001, − 0.000) 0.025	− 0.000 (− 0.001, 0.001) 0.488	− 0.000 (− 0.001, 0.000) 0.392
Age 45–60	− 0.002 (− 0.003, − 0.001) 0.002	− 0.001 (− 0.003, − 0.000) 0.026	− 0.001 (− 0.002, 0.000) 0.179
Age ≥ 60	− 0.002 (− 0.003, − 0.000) 0.013	− 0.002 (− 0.003, − 0.000) 0.015	− 0.001 (− 0.002, 0.000) 0.227

Logistic regression models: Model 1: no covariates were adjusted. Model 2 was adjusted for demographic factors, including gender, age, race. Model 3 was adjusted for gender, age, race, education, smoking behavior, alcohol consumption, BMI, HbA1c, albumin, blood urea nitrogen, total cholesterol, triglycerides, total calcium, creatinine, phosphorus, uric acid

### The subgroup analyses were stratified by gender and age

**Fig. 2:**
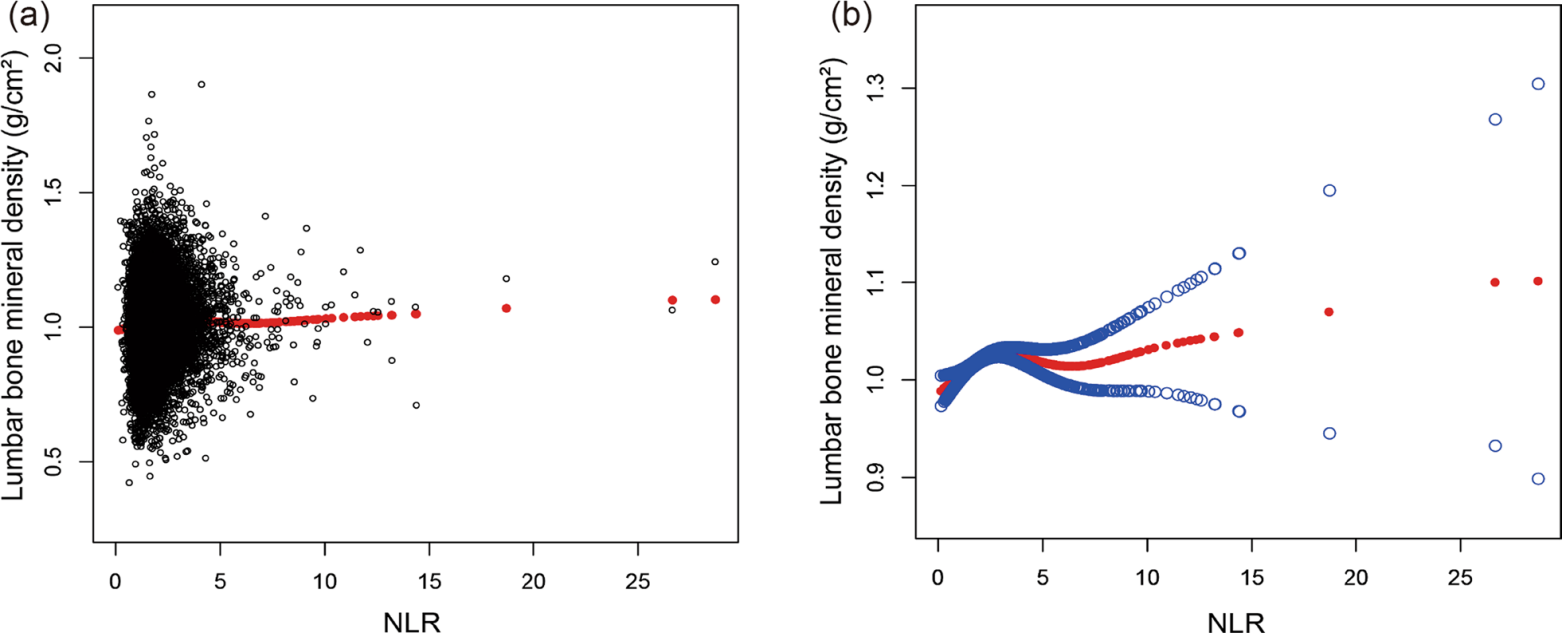
The association NLR between and lumbar BMD. **a** Each black point represents a sample. **b** Solid rad line represents the smooth curve fit between variables. Blue bands represent the 95% of confidence interval from the fit. Age, gender, race, education, smoking behavior, alcohol consumption, BMI, HbA1c, albumin, blood urea nitrogen, total cholesterol, triglycerides, total calcium, creatinine, phosphorus, and uric acid were adjusted

**Fig. 3:**
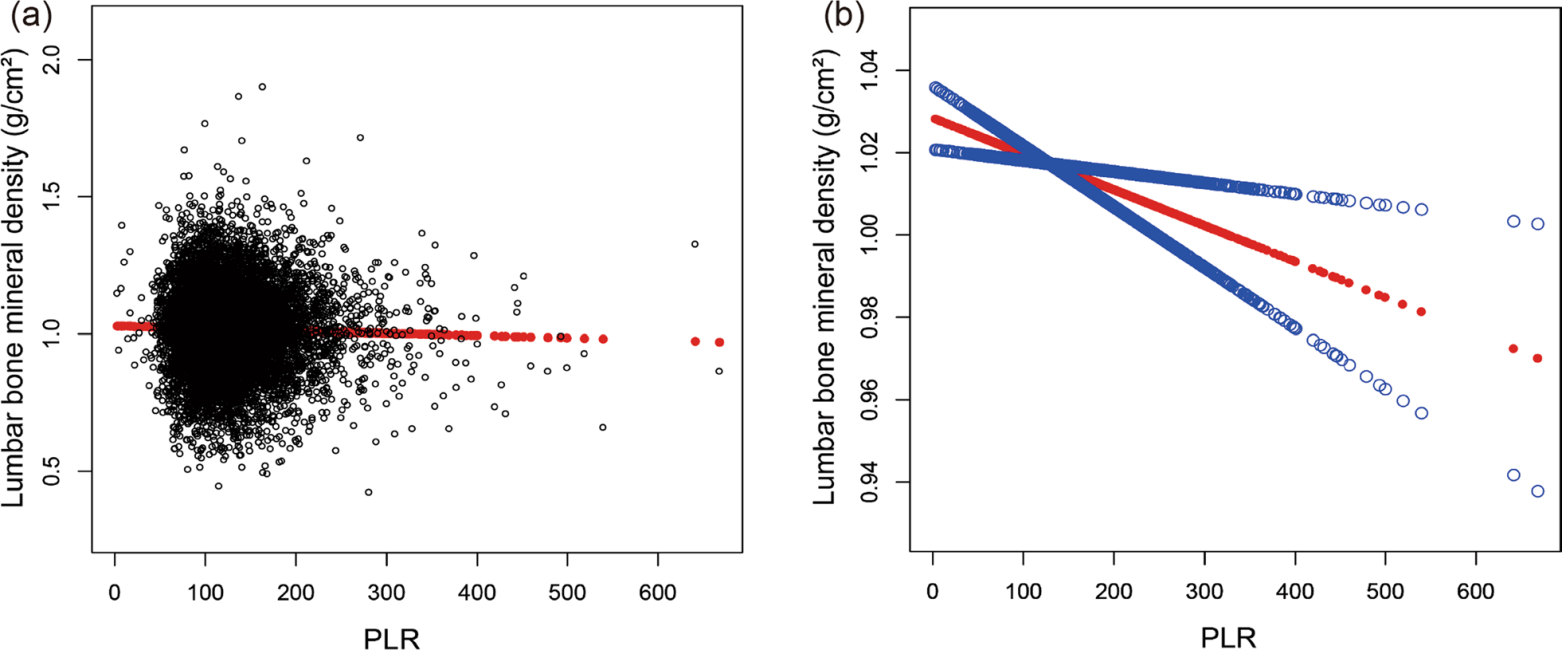
The association PLR between and lumbar BMD. **a** Each black point represents a sample. **b** Solid rad line represents the smooth curve fit between variables. Blue bands represent the 95% of confidence interval from the fit. Age, gender, race, education, smoking behavior, alcohol consumption, BMI, HbA1c, albumin, blood urea nitrogen, total cholesterol, triglycerides, total calcium, creatinine, phosphorus, and uric acid were adjusted

**Fig. 4:**
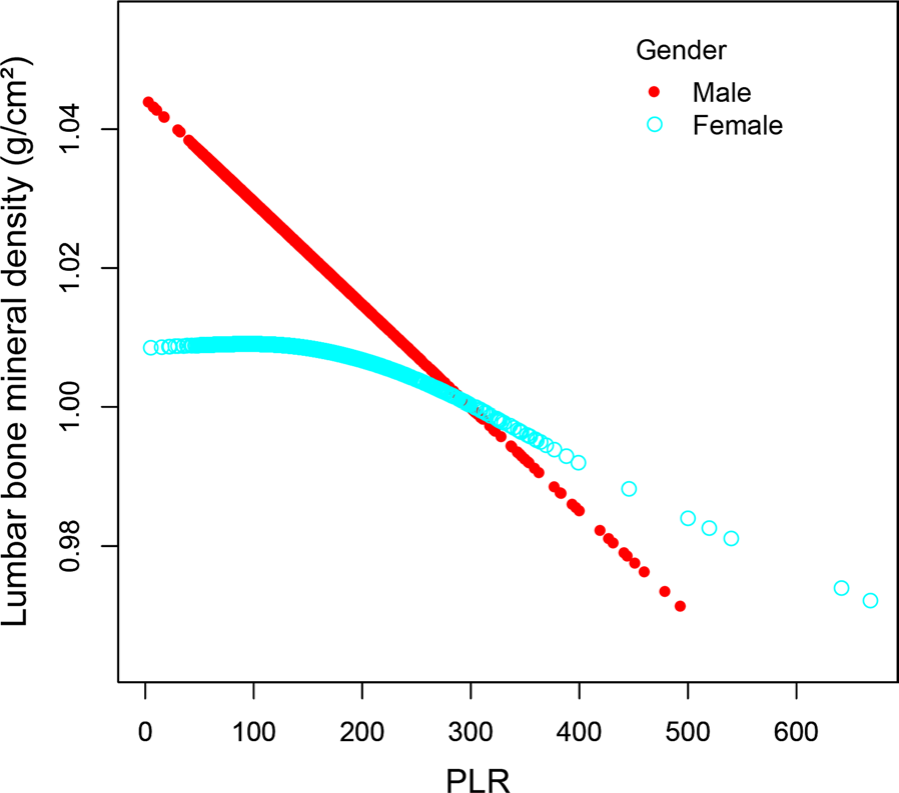
The association between PLR and lumbar bone mineral density stratified by gender. Age, race, education, smoking behavior, alcohol consumption, BMI, HbA1c, albumin, blood urea nitrogen, total cholesterol, triglycerides, total calcium, creatinine, phosphorus, and uric acid were adjusted

**Fig. 5:**
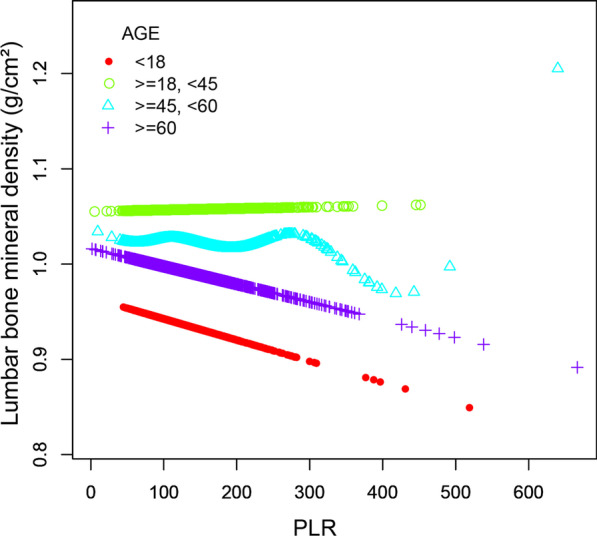
The association between PLR and lumbar bone mineral density stratified by age. Gender, race, education, smoking behavior, alcohol consumption, BMI, HbA1c, albumin, blood urea nitrogen, total cholesterol, triglycerides, total calcium, creatinine, phosphorus, and uric acid were adjusted

As presented in Figs. 2 and 3, NLR was positively associated with lumbar spine BMD on the whole, and PLR was negatively linearly correlated with lumbar BMD. Further analyses were conducted on subgroups of gender and age to verify the robustness of the correlation between PLR levels and lumbar BMD. The subgroup analyses stratified by gender and age were shown in Figs. 4 and 5, which show negative relationships between PLR and lumbar spine BMD in male and age < 18 groups were consistently significant after adjusting for different covariates.

## Discussion

In the current study, we explored the association between inflammatory markers and BMD using the NHANES 2007–2010 data. This study found that higher NLR levels and lower PLR levels were associated with higher lumbar BMD, whereas MLR was not significantly linked to lumbar spine BMD. In subgroup analyses stratified by gender and age, the strong relationship between PLR and lumbar BMD was observed in the male and age < 18 groups. To our knowledge, this is the first cross-sectional study in the USA to study correlations between inflammatory markers and BMD, revealing the close association between chronic inflammation and bone metabolism.

Accumulating evidence has confirmed a close correlation between chronic inflammation and bone remodeling, which might be related to age-related oxidative stress and the activation of the immune system [26]. Aging could keep the immune system in a state of constant subclinical inflammatory, affecting the T and B lymphocytes to varying degrees, thereby breaking the balance of inflammatory factors and protective immunity factors in bone metabolism [27]. IL-1, IL-9, TNF-αand other inflammatory mediators have been reported to promote osteoclasts (OCs) differentiation or inhibit bone marrow mesenchymal stem cells (MSCs) osteogenic differentiation through the activation of the RANKL/RANK/OPG pathway and Wnt signaling pathway [28]. Dr. Stojanovic and colleagues conducted a prospective population-based study among older adults and observed that higher baseline levels of IL-6 and white blood cell (WBC) were linked to a higher incidence of hip fractures [29]. Moreover, according to Monaco et al., total lymphocyte count was significantly positively correlated with femoral BMD in healthy postmenopausal women [30].

A number of recent studies have revealed that blood routine examination-derived biomarkers, such as MLR, NLR, and PLR, whose might be closely associated with systemic inflammation and immune response status [31, 32]. Due to these composite inflammation indices being calculated from multiple inflammatory cell counts according to the formula, they have the advantages of being easy to measure and relatively stable compared with other inflammatory markers such as IL-6, IL-9 and TNF-α [19, 33]. Only peripheral blood samples are required, which almost cause no discomfort. Therefore, these novel inflammatory indices have attracted a great deal of research interest. To our knowledge, NLR and PLR have been confirmed to be significantly related to the occurrence and development of coronary syndrome, sarcopenia, rheumatoid arthritis and other diseases [34–37], but their value in osteoporosis remains to be firmly established due to the lack of evidence.

In the present study, the results revealed that NLR, PLR increased and decreased prominently in participants with higher lumbar BMD, respectively. And the association between PLR and BMD in the lumbar spine, total femur, and femoral neck remained significant. Similarly, Yilmaz et al. [38] reported that levels of NLR and CRP in the osteoporosis group were higher than those in the bone healthy group, and it was emphasized that NLR could be a more reliable indicator of osteoporosis than CRP in postmenopausal women. In a clinical study of 211 postmenopausal women, Koseoglu et al. [39] illustrated the correlation between PLR levels and lumbar BMD was significant negative, and PLR levels decreased with increasing osteoporosis severity. The different predictive effects of NLR and PLR might be caused by the different effects of platelets and neutrophils. In chronic inflammation, neutrophil over-activation promotes osteoblast apoptosis by releasing reactive oxygen species and increasing osteoclastogenesis through RANKL signaling [21]. PLT are megakaryocyte-derived cytoplasmic fragments that play important roles in bone homeostasis, bone formation, and bone resorption [40, 41]. Several studies have highlighted the supportive effects of PLT on bone formation, in which platelet-derived growth factors (PDGFs) favor bone formation by influencing cell proliferation, chemotaxis differentiation, and extracellular matrix synthesis [42]. Therefore, we speculated that in the case of bone loss, our body needs more platelet rather than lymphocytes. The ratio of platelet count to lymphocyte count will increase when platelets increase and lymphocytes decrease. This is one of the reasons why low BMD is significantly associated with increased PLR levels. However, the exact mechanisms are needed to be confirmed in future studies.

In addition, some information in the subgroup analysis should also be noted. First of all, in the subgroup analysis by gender, we discovered a significant negative relationship between PLR levels and lumbar BMD in males, but not in females. Likewise, several studies have discovered that inflammatory markers have higher diagnostic value in gastric cancer, myocardial fibrosis, and osteoporosis in males compared to females [43–45]. On the one hand, males and females had different susceptibility to inflammation and sexual dimorphism was observed in inflammation-related conditions all along the life course [46, 47]. On the other hand, male immune systems reacted more strongly to inflammatory factors entering the peripheral blood, leading to an increase in neutrophils and platelets [44]. Moreover, our results also indicated that PLR levels and lumbar BMD are related in different age groups, suggesting this relationship is independent of age. Nevertheless, the association was not significant after adjusting for multiple factors. This might be due to differences in genetic risk factors, obesity status, lifestyle habits, and other confounding factors.

Our study has some strengths. To begin with, we utilized the generalizability of NHANES data, which contained representative non-institutionalized Americans, which allowed our findings to be presented as generalizable. Second, we adjusted for covariates, such as demographic factors, lifestyle habits, and laboratory indices. In this way, potential sources of bias could be minimized. Last but not least, multiple logistic regression and the stratified analysis were used to provide reliable evidence of an independent relationship between PLR and BMD. However, we are also aware of several limitations of our study. Firstly, due to the lack of anti-osteoporotic treatment information, the objectivity of BMD measurements might be compromised. Besides, the information on medical history, smoking behavior, and alcohol consumption was self-reported data obtained from questionnaires; thus, recall bias and several other errors could not be ruled out. Finally, for the cross-sectional design of NHANES, we could not investigate the causal association between inflammatory markers and BMD.

## Conclusions

The present study confirms that MLR was not linked to lumbar spine BMD, while NLR and PLR are positively and negatively correlated with lumbar BMD, respectively. And PLR is considered an effective indicator of the complex relationship between chronic inflammation and bone loss. However, further prospective cohort studies and mechanistic studies are needed to confirm these findings.

## Supplementary Information

**Additional file 1.** Data analysis code.

## Data Availability

The survey data are publicly available on the internet for data users and researchers throughout the world http://www.cdc.gov/nchs/nhanes/.

## Abbreviations

BMD: Bone density mineral
BMI: Body mass index
NHANES: The National Health and Nutrition Examination Survey
CRP: C-reactive protein
IL-1: Interleukin-1
IL-6: Interleukin-6
TNF-α: Tumor Necrosis Factor-α
MLR: Monocyte-to-lymphocyte ratio
NLR: Neutrophil-to-lymphocyte ratio
PLR: Platelet-to-lymphocyte ratio
FBC: Full blood count
TC: Total cholesterol
P: Phosphorus
HDL-C: High-density lipoprotein cholesterol
BUN: Urea nitrogen
TG: Triglycerides
Hb: Hemoglobin
PLT: Platelet count
OCs: Osteoclasts
MSCs: Mesenchymal stem cells
WBC: White blood cell
PDGFs: Platelet-derived growth factors
