# Supplementary material for: Association between inflammatory markers and bone mineral density: a cross-sectional study from NHANES 2007–2010
Source: J Orthop Surg Res. 2023 Apr 17;18:305. doi: 10.1186/s13018-023-03795-5 (PMC10108543; doi:10.1186/s13018-023-03795-5)
Supplement: Supplementary file 1 — Additional file 1. Data analysis code. [file 13018_2023_3795_MOESM1_ESM.docx]

**Data analysis code**

library(doBy,lib.loc=R.LibLocation)

library(plotrix,lib.loc=R.LibLocation)

library(stringi,lib.loc=R.LibLocation)

library(stringr,lib.loc=R.LibLocation)

library(survival,lib.loc=R.LibLocation)

library(rms,lib.loc=R.LibLocation)

library(nnet,lib.loc=R.LibLocation)

library(car,lib.loc=R.LibLocation)

library(mgcv,lib.loc=R.LibLocation)

pdfwd<-6; pdfht<-6

setwd("D:/Nhanes/shuju/PROJ11_1_tbl")

load("D:/Nhanes/shuju/XUE07101.Rdata")

if (length(which(ls()=="EmpowerStatsR"))==0) EmpowerStatsR<-get(ls()[1])

names(EmpowerStatsR)<-toupper(names(EmpowerStatsR))

rankvar <- function(var, num) {

qprobs <- 1/num

if (num>2) {for (i in (2:(num-1))) {qprobs <- c(qprobs, 1/num * i) }}

outvar <- rep(0, times=length(var))

outvar[is.na(var)] <- NA

cutpoints <- quantile(var,probs=qprobs, na.rm=TRUE)

for (k in (1:length(cutpoints))) {outvar[var>=cutpoints[k]] <- k}

tmp<-c(min(var,na.rm=TRUE),cutpoints,max(var,na.rm=TRUE))

names(tmp)<-c("Min",names(cutpoints),"Max")

print(tmp)

return(outvar)

}

attach(EmpowerStatsR)

sink("D:/Nhanes/shuju/datastep/PROJ11_datastep.lst")

print("Creating new variable: BMD.Q4")

BMD.Q4<- rankvar(BMD,4)

EmpowerStatsR<-cbind(EmpowerStatsR,BMD.Q4)

print("Creating new variable: PLR.Q4")

PLR.Q4<- rankvar(PLR,4)

EmpowerStatsR<-cbind(EmpowerStatsR,PLR.Q4)

print("Creating new variable: AGE.CS")

AGE.CS<- 0+(AGE>=18)+(AGE>=45)+(AGE>=60)

AGE.CS[is.na(AGE)]<-NA

EmpowerStatsR<-cbind(EmpowerStatsR,AGE.CS)

rm(BMD.Q4,PLR.Q4,AGE.CS)

detach(EmpowerStatsR)

sink()

vname<-c(vname,"RED","HB","PLT","NLR","PLR","LMR","SII","ALQ101","ALQ101.1","ALQ101.2","ALQ101.3")

vlabel<-c(vlabel,"RED","HB","PLT","NLR","PLR","LMR","SII","ALQ101"," 1"," 2"," 3")

vname<-c(vname,"BP","BP.1","BP.2","BP.3","BMI","HBAIC","GENE","GENE.1","GENE.2")

vlabel<-c(vlabel,"BP"," 1"," 2"," 3","BMI","HBAIC","GENE"," 1"," 2")

vname<-c(vname,"AGE","RACE","RACE.1","RACE.2","RACE.3","RACE.4")

vlabel<-c(vlabel,"AGE","RACE"," 1"," 2"," 3"," 4")

vname<-c(vname,"DMDEDUC1","DMDEDUC1.1","DMDEDUC1.2","DMDEDUC1.3","DMDEDUC1.4")

vlabel<-c(vlabel,"DMDEDUC1"," 1"," 2"," 3"," 4")

vname<-c(vname,"WTINT2YR","WTMEC2YR","PHYSICAL","PHYSICAL.1","PHYSICAL.2","PHYSICAL.9")

vlabel<-c(vlabel,"WTINT2YR","WTMEC2YR","PHYSICAL"," 1"," 2"," 9")

vname<-c(vname,"SMOKE","SMOKE.1","SMOKE.2","SMOKE.3","SPORT","SPORT.1","SPORT.2","SPORT.3")

vlabel<-c(vlabel,"SMOKE"," 1"," 2"," 3","SPORT"," 1"," 2"," 3")

vname<-c(vname,"DIQ","DIQ.1","DIQ.2","DIQ.3","FBMD","NKBMD")

vlabel<-c(vlabel,"DIQ"," 1"," 2"," 3","FBMD","NKBMD")

vname<-c(vname,"TRBMD","INBMD","WDBMD","BMD")

vlabel<-c(vlabel,"TRBMD","INBMD","WDBMD","BMD")

vname<-c(vname,"ALBUMIN","UREA","CA","TC")

vlabel<-c(vlabel,"ALBUMIN","UREA","CA","TC")

vname<-c(vname,"CREATININE","P","TG","URIC.ACID","BMD.Q4","BMD.Q4.0","BMD.Q4.1","BMD.Q4.2","BMD.Q4.3")

vlabel<-c(vlabel,"CREATININE","P","TG","URIC.ACID","BMD (quartiles)"," Q1"," Q2"," Q3"," Q4")

vname<-c(vname,"PLR.Q4","PLR.Q4.0","PLR.Q4.1","PLR.Q4.2","PLR.Q4.3")

vlabel<-c(vlabel,"PLR (quartiles)"," Q1"," Q2"," Q3"," Q4")

vname<-c(vname,"AGE.CS","AGE.CS.0","AGE.CS.1","AGE.CS.2","AGE.CS.3")

vlabel<-c(vlabel,"AGE (quartiles)"," <18"," >=18, <45"," >=45, <60"," >=60")

slt.vname<-c()

library(weights,lib.loc=R.LibLocation)

library(Hmisc,lib.loc=R.LibLocation)

ofname<-"PROJ11_1_tbl";

WD<-EmpowerStatsR; wd.subset="";

svy.DSN.YN <- FALSE;

weights<-WD$WTINT2YR;weights.var <- 'wtint2yr';

WD<-cbind(WD,weights); WD<-WD[!is.na(weights),];

title<-"Table 1";

attach(WD)

subjvname<-NA;

xv<-cbind(GENE,AGE,RACE,DMDEDUC1,SMOKE,DIQ,BMI,HBAIC,ALQ101,ALBUMIN,UREA,CA,TC,CREATININE,P,TG,URIC.ACID,L,M,N,RED,HB,PLT,NLR,PLR,LMR,SII);

xvname<-c('GENE','AGE','RACE','DMDEDUC1','SMOKE','DIQ','BMI','HBAIC','ALQ101','ALBUMIN','UREA','CA','TC','CREATININE','P','TG','URIC.ACID','L','M','N','RED','HB','PLT','NLR','PLR','LMR','SII');

xvar<-c('GENE','AGE','RACE','DMDEDUC1','SMOKE','DIQ','BMI','HBAIC','ALQ101','ALBUMIN','UREA','CA','TC','CREATININE','P','TG','URIC_ACID','L','M','N','RED','HB','PLT','NLR','PLR','LMR','SII');

xlv<-c(2,0,4,4,3,3,0,0,3,0,0,0,0,0,0,0,0,0,0,0,0,0,0,0,0,0,0);

sxf<-NA;

svname<-NA; sv<-NA; slv<-NA;

av<-NA; avname<-NA; avlbl<-NA; nadj<-0; alv<-NA;

timev<-NA; timevname<-NA;

bv<-BMD.Q4;bvar<-"BMD.Q4";bvname<-"BMD_Q4";

colv<-NA; colvname<-NA;

v.start<-NA; vname.start<-NA;

v.stop<-NA; vname.stop<-NA;

par1<-NA;dec<-2;parm<-c(NA, NA, 1,NA, 0);

if (!exists("pdfwd")) pdfwd<-6;

if (!exists("pdfht")) pdfht<-6;

##R package## weights Hmisc ##R package##;

mat2htmltable<-function(mat) {

t1<- apply(mat,1,function(z) paste(z,collapse="</td><td>"))

t2<- paste("<tr><td>",t1,"</td></tr>")

return(paste(t2,collapse=" "))

}

pvformat<-function(p,dec) {

pp <- sprintf(paste("%.",dec,"f",sep=""),as.numeric(p))

if (is.matrix(p)) {pp<-matrix(pp, nrow=nrow(p)); colnames(pp)<-colnames(p);rownames(pp)<-rownames(p);}

lw <- paste("<",substr("0.00000000000",1,dec+1),"1",sep="");

pp[as.numeric(p)<(1/10^dec)]<-lw

return(pp)

}

numfmt<-function(p,dec) {

if (is.list(p)) p<-as.matrix(p)

pp <- sprintf(paste("%.",dec,"f",sep=""),as.numeric(p))

if (is.matrix(p)) {pp<-matrix(pp, nrow=nrow(p));colnames(pp)<-colnames(p);rownames(pp)<-rownames(p);}

pp[as.numeric(p)>10000000]<- "inf."

pp[is.na(p) | gsub(" ","",p)==""]<- ""

pp[p=="-Inf"]<-"-Inf"

pp[p=="Inf"]<-"Inf"

return(pp)

}

lmp <- function (mdl, dec) {

p <- NA

if (class(mdl) == "lm") {f <- summary(mdl)$fstatistic; p <- pvformat(pf(f[1],f[2],f[3],lower.tail=F),dec); }

return(p);

}

vlabelN<-(substr(vlabel,1,1)==" ");

vlabelZ<-vlabel[vlabelN];vlabelV<-vlabel[!vlabelN]

vnameV<-vname[!vlabelN];vnameZ<-vname[vlabelN];

w<-c("<html><head>","<meta http-equiv=\"Content-Type\" content=\"text/html\" charset=\"gb2312\" /></head><body>")

w<-c(w,paste("<h2>", title, "</h2>"))

allvname<-c(xvname,bvar,colvname,"weights"); allvname<-allvname[!is.na(allvname)];

WD<-data.frame(WD,TOT_=1)[,c(allvname,"TOT_")];

rm(xv,bv,colv)

decp <- max(dec+2,4);

if (is.na(colvname)) {

nclv<-1; clvb<-"Total"; clvb_<-"Total"

} else {

clv<-levels(factor(WD[,colvname])); nclv<-length(clv)+1

clvb_<-vlabelZ[match(paste(colvname,".",clv,sep=""),vnameZ)];

clvb_[is.na(clvb_)]<-clv[is.na(clvb_)];

clvb<-c(paste(vlabelV[vnameV==colvname],clvb_,sep="="),"Total");

clvb_<-c(clvb_,"Total")

WD<-WD[!is.na(WD[,colvname]),]

}

if (is.na(bvar)) {ncc<-1; tt00<-"";

} else {

bvb<-vlabelV[vnameV==bvar];

blv<-levels(factor(WD[,bvar])); ncc<-length(blv);

blvb_<-vlabelZ[match(paste(bvar,".",blv,sep=""),vnameZ)];

blvb_[is.na(blvb_)]<-blv[is.na(blvb_)];

tt00<-c(blvb_,"P-value","P-value*")

WD<-WD[!is.na(WD[,bvar]),];

}

xv0<-xvname[xlv==0]; xv1<-xvname[xlv>0]; nxv0<-sum(xlv==0); nxv1<-sum(xlv>0);

xv0b<-vlabelV[match(xv0,vnameV)]; xv0b[is.na(xv0b)]<-xv0[is.na(xv0b)]

xv1b<-vlabelV[match(xv1,vnameV)]; xv1b[is.na(xv1b)]<-xv1[is.na(xv1b)]

for (k in 1:nclv) {

if (!is.na(colvname) & k<nclv) {WD1<-WD[WD[,colvname]==clv[k],]; } else {WD1<-WD;}

if (!is.na(bvar)) {

tt <- c("", blvb_, "P value");

if (ncc==2) tt <- c("", blvb_, "Standardize diff.", "P value");

if (nxv0>0) {

tt0 <- c("",xv0b);

meanxx<-matrix(NA, nrow=nxv0, ncol=ncc);

stdxx <-matrix(NA, nrow=nxv0, ncol=ncc);

nnxx <-matrix(NA, nrow=nxv0, ncol=ncc);

for (i in (1:ncc)) {

coli <- blvb_[i];

WD2 <- WD1[WD1[,bvar]== blv[i],]

for (j in (1:nxv0)) {

meani <- wtd.mean(WD2[,xv0[j]], WD2$weights, na.rm=TRUE)

stdi <- sqrt(wtd.var(WD2[,xv0[j]], WD2$weights, na.rm=TRUE))

coli <- c(coli, paste(numfmt(meani,dec)," &#177 ", numfmt(stdi,dec), sep=""))

meanxx[j,i]<-meani; stdxx[j,i]<-stdi

nnxx[j,i] <- sum(WD2$weights, na.rm=TRUE)

}

tt0 <- cbind(tt0, coli);

}

if (ncc==2) {

st.diff<-""

for (j in 1:nxv0) {

stddiff <- abs(meanxx[j,2] - meanxx[j,1])/sqrt((stdxx[j,2]^2 + stdxx[j,1]^2)/2)

se <- sqrt((nnxx[j,1]+nnxx[j,2])/nnxx[j,1]/nnxx[j,2] + stddiff^2/(2*(nnxx[j,1]+nnxx[j,2])))

stddiff.l <- stddiff - 1.96 * se

stddiff.u <- stddiff + 1.96 * se

vi.stdiff<-paste(numfmt(stddiff,3), " (", numfmt(stddiff.l,3), ", ", numfmt(stddiff.u,3), ")", sep="")

st.diff<-c(st.diff,vi.stdiff)

}

tt0 <- cbind(tt0, st.diff)

}

colp <- "P-value";

for (j in 1:nxv0) {

mdl<- lm(WD1[,xv0[j]]~factor(WD1[,bvar]), weights=WD1$weights, data=WD1)

colp <- c(colp, lmp(mdl, decp))

}

tt0 <-cbind(tt0,colp);

tt <- rbind(tt, tt0[-1,])

}

if (nxv1>0) {

tt1 <- c("", blvb_, "P value");

if (ncc==2) tt1 <- c("", blvb_, "Standardize diff.", "P value");

for (j in (1:nxv1)) {

vlv<-levels(factor(WD1[,xv1[j]]));

vlvb_<-vlabelZ[match(paste(xv1[j],".",vlv,sep=""),vnameZ)];

vlvb_[is.na(vlvb_)]<-vlv[is.na(vlvb_)];

colxj <- c(xv1b[j],vlvb_);

z <- NULL; sumw <- NULL

for (i in (1:ncc)) {

WD2 <- WD1[WD1[,bvar]== blv[i],]

freqi <- wpct(WD2[,xv1[j]],weight=WD2$weights,na.rm=TRUE)

ffi <- freqi[match(vlv,names(freqi))]

z <-cbind(z, ffi)

ffi <- c("",numfmt(freqi*100,dec))

colxj <-cbind(colxj, ffi)

sumw <- c(sumw, sum(WD2$weights,na.rm=TRUE))

}

if (ncc==2) {

stddiff <- abs(z[,1]-z[,2])/sqrt((z[,1]*(1-z[,1])+z[,2]*(1-z[,2]))/2)

se<-sqrt((sumw[1]+sumw[2])/sumw[1]/sumw[2] + stddiff^2/(2*(sumw[1]+sumw[2])))

stddiff.l <- stddiff - 1.96 * se

stddiff.u <- stddiff + 1.96 * se

vi.stdiff<-paste(numfmt(stddiff,3), " (", numfmt(stddiff.l,3), ", ", numfmt(stddiff.u,3), ")", sep="")

colxj <- cbind(colxj, c("", vi.stdiff))

}

chi <- wtd.chi.sq(WD1[,xv1[j]],WD1[,bvar],weight=WD1$weights,na.rm=TRUE)

pv <- chi["p.value"];

colp <- c(pvformat(pv,decp), rep("",times=length(vlv)))

colxj<- cbind(colxj, colp)

tt1 <- rbind(tt1, colxj)

}

tt <- rbind(tt, tt1[-1,])

}

} else {

tt <- c("", "Statistics");

if (nxv0>0) {

tt0 <- c("",xv0b);

colxx = "";

for (j in (1:nxv0)) {

meani <- wtd.mean(WD1[,xv0[j]], WD1$weights, na.rm=TRUE)

stdi <- sqrt(wtd.var(WD1[,xv0[j]], WD1$weights, na.rm=TRUE))

colxx <- c(colxx, paste(numfmt(meani,dec)," &#177 ", numfmt(stdi,dec), sep=""))

}

tt0<-cbind(tt0,colxx)

tt <- rbind(tt, tt0[-1,])

}

if (nxv1>0) {

tt1 <- c("", "%");

for (j in (1:nxv1)) {

vlv<-levels(factor(WD1[,xv1[j]]));

vlvb_<-vlabelZ[match(paste(xv1[j],".",vlv,sep=""),vnameZ)];

vlvb_[is.na(vlvb_)]<-vlv[is.na(vlvb_)];

colxj <- c(xv1b[j],vlvb_);

freqi <- wpct(WD1[,xv1[j]],weight=WD1$weights,na.rm=TRUE)

ffi <- freqi[match(vlv,names(freqi))]

ffi <- c("",numfmt(freqi*100,dec))

colxj <-cbind(colxj, ffi)

tt1 <- rbind(tt1, colxj)

}

tt <- rbind(tt, tt1[-1,])

}

}

if (!is.na(colvname)) w<-c(w,"</br>",clvb[k])

w<-c(w,"</br><table border=3>", mat2htmltable(tt), "</table>")

}

if (nxv0>0) {

w<-c(w,"</br>Mean +/- SD for: ", paste(xv0b,sep="; "))

if (!is.na(bvar)) w<-c(w, ". P value was calculated by weighted linear regression model.");

}

if (nxv1>0) {

w<-c(w,"</br> % for: ", paste(xv1b,sep="; "))

if (!is.na(bvar)) w<-c(w, ". P value was calculated by weighted chi-square test.");

}

w<-c(w,paste("</br>Created by EmpowerStats (www.empowerstats.com) and R on",Sys.Date()))

w<-c(w,wd.subset)

w<-c(w,"</body></html>")

fileConn<-file(paste(ofname,".htm",sep="")); writeLines(w, fileConn)

library(gdata,lib.loc=R.LibLocation)

library(geepack,lib.loc=R.LibLocation)

library(mgcv,lib.loc=R.LibLocation)

ofname<-"PROJ11_2_tbl1";

WD<-EmpowerStatsR; wd.subset="";

svy.DSN.YN <- FALSE;

weights<-WD$WTINT2YR;weights.var <- 'wtint2yr';

WD<-cbind(WD,weights); WD<-WD[!is.na(weights),];

title<-"Table 2";

attach(WD)

subjvname<-NA;

yv<-cbind(BMD);

yvname<-c('BMD');

yvar<-c('BMD');

ydist<-c('gaussian');

ylink<-c('identity');

ylv<-c(0);

xv<-cbind(PLT,NLR,PLR,PLR.Q4);

xvname<-c('PLT','NLR','PLR','PLR.Q4');

xvar<-c('PLT','NLR','PLR','PLR_Q4');

xlv<-c(0,0,0,4);

sxf<-c(NA,0,0,0,0)[-1];

sv<-cbind(GENE,AGE,RACE,DMDEDUC1,BP,BMI,HBAIC,ALQ101,DIQ,SMOKE,UREA,CA,CREATININE,P,TG,URIC.ACID);

svname<-c('GENE','AGE','RACE','DMDEDUC1','BP','BMI','HBAIC','ALQ101','DIQ','SMOKE','UREA','CA','CREATININE','P','TG','URIC.ACID');

svar<-c('GENE','AGE','RACE','DMDEDUC1','BP','BMI','HBAIC','ALQ101','DIQ','SMOKE','UREA','CA','CREATININE','P','TG','URIC_ACID');

sdf<-c(NA,0,0,0,0,0,0,0,0,0,0,0,0,0,0,0,0)[-1];

slv<-c(2,0,4,4,3,0,0,3,3,3,0,0,0,0,0,0);

av<-cbind(GENE,AGE,RACE);

avname<-c('GENE','AGE','RACE');

if (!is.na(avname[1])) avlbl<-vlabel[match(avname, vname)];

nadj<-length(avname);alv<-c(2,0,4);

saf<-c(NA,0,0,0)[-1];

timev<-NA; timevname<-NA;

bv<-NA; bvar<-NA;

colv<-NA; colvname<-NA;

v.start<-NA; vname.start<-NA;

v.stop<-NA; vname.stop<-NA;

par1<-1;dec<-4;parm<-c(1,NA, 1,1, 0);

if (!exists("pdfwd")) pdfwd<-6;

if (!exists("pdfht")) pdfht<-6;

##R package## gdata geepack mgcv ##R package##;

pvformat<-function(p,dec) {

pp <- sprintf(paste("%.",dec,"f",sep=""),as.numeric(p))

if (is.matrix(p)) {pp<-matrix(pp, nrow=nrow(p)); colnames(pp)<-colnames(p);rownames(pp)<-rownames(p);}

lw <- paste("<",substr("0.00000000000",1,dec+1),"1",sep="");

pp[as.numeric(p)<(1/10^dec)]<-lw

return(pp)

}

numfmt<-function(p,dec) {

if (is.list(p)) p<-as.matrix(p)

pp <- sprintf(paste("%.",dec,"f",sep=""),as.numeric(p))

if (is.matrix(p)) {pp<-matrix(pp, nrow=nrow(p));colnames(pp)<-colnames(p);rownames(pp)<-rownames(p);}

pp[as.numeric(p)>10000000]<- "inf."

pp[is.na(p) | gsub(" ","",p)==""]<- ""

pp[p=="-Inf"]<-"-Inf"

pp[p=="Inf"]<-"Inf"

return(pp)

}

varstats<-function(var,vlvl,dec) {

if (length(vlvl)==1 & vlvl[1]==0) {

return(paste(numfmt(mean(var,na.rm=TRUE),dec),numfmt(sd(var,na.rm=TRUE),dec),sep="+"))

} else {

a<-table(var)

b<-matrix(paste(a, " (", numfmt(a/sum(a)*100,dec), "%)",sep=""),ncol=1)

return(c(" ",b[match(vlvl,names(a))]))

}

}

mat2htmltable<-function(mat) {

t1<- apply(mat,1,function(z) paste(z,collapse="</td><td>"))

t2<- paste("<tr><td>",t1,"</td></tr>")

return(paste(t2,collapse=" "))

}

setgam<-function(fml,yi) {

if (ydist[yi]=="") ydist[yi]<-"gaussian"

if (ydist[yi]=="exact") ydist[yi]<-"binomial"

if (ydist[yi]=="breslow") ydist[yi]<-"binomial"

if (ydist[yi]=="gaussian") mdl<-try(gam(formula(fml),weights=wdtmp$weights,data=wdtmp, family=gaussian(link="identity")))

if (ydist[yi]=="binomial") mdl<-try(gam(formula(fml),weights=wdtmp$weights,data=wdtmp, family=binomial(link="logit")))

if (ydist[yi]=="poisson") mdl<-try(gam(formula(fml),weights=wdtmp$weights,data=wdtmp, family=poisson(link="log")))

if (ydist[yi]=="gamma") mdl<-try(gam(formula(fml),weights=wdtmp$weights,data=wdtmp, family=Gamma(link="inverse")))

if (ydist[yi]=="negbin") mdl<-try(gam(formula(fml),weights=wdtmp$weights,data=wdtmp, family=negbin(c(1,10), link="log")))

return(mdl)

}

setgee<-function(fml,yi) {

if (ydist[yi]=="") ydist[yi]<-"gaussian"

if (ydist[yi]=="exact") ydist[yi]<-"binomial"

if (ydist[yi]=="breslow") ydist[yi]<-"binomial"

if (ydist[yi]=="gaussian") md<-try(geeglm(formula(fml),id=wdtmp[,subjvname],corstr=gee.TYPE,family="gaussian",weights=wdtmp$weights,data=wdtmp))

if (ydist[yi]=="binomial") md<-try(geeglm(formula(fml),id=wdtmp[,subjvname],corstr=gee.TYPE,family="binomial",weights=wdtmp$weights,data=wdtmp))

if (ydist[yi]=="poisson") md<-try(geeglm(formula(fml),id=wdtmp[,subjvname],corstr=gee.TYPE,family="poisson",weights=wdtmp$weights,data=wdtmp))

if (ydist[yi]=="gamma") md<-try(geeglm(formula(fml),id=wdtmp[,subjvname],corstr=gee.TYPE,family="Gamma",weights=wdtmp$weights,data=wdtmp))

if (ydist[yi]=="negbin") md<-try(geeglm.nb(formula(fml),id=wdtmp[,subjvname],corstr=gee.TYPE,weights=wdtmp$weights,data=wdtmp))

return(md)

}

setglm<-function(fml,yi) {

if (ydist[yi]=="") ydist[yi]<-"gaussian"

if (ydist[yi]=="exact") ydist[yi]<-"binomial"

if (ydist[yi]=="breslow") ydist[yi]<-"binomial"

if (ydist[yi]=="gaussian") md<-try(glm(formula(fml),family="gaussian",weights=wdtmp$weights,data=wdtmp))

if (ydist[yi]=="binomial") md<-try(glm(formula(fml),family="binomial",weights=wdtmp$weights,data=wdtmp))

if (ydist[yi]=="poisson") md<-try(glm(formula(fml),family="poisson",weights=wdtmp$weights,data=wdtmp))

if (ydist[yi]=="gamma") md<-try(glm(formula(fml),family="Gamma",weights=wdtmp$weights,data=wdtmp))

if (ydist[yi]=="negbin") md<-try(glm.nb(formula(fml),weights=wdtmp$weights,data=wdtmp))

return(md)

}

mdl2oo<-function(mdl, xxname, opt) {

if (is.na(mdl[[1]][1])) return(list(rep("",times=length(xxname)),""))

if (substr(mdl[[1]][1],1,5)=="Error") return(list(rep("",times=length(xxname)),""))

gs<-summary(mdl); print(mdl$formula); print(gs)

if (opt=="gam") {gsparm <- gs$p.table;tmpn<-gs$n;

} else {gsparm <- gs$coefficients;tmpn <- sum(gs$df[c(1,2)]);}

gsp<-gsparm[match(xxname,rownames(gsparm)),]

if (length(xxname)==1) {beta<-gsp[1]; se<-gsp[2]; pv<-gsp[4];

} else {beta<-gsp[,1]; se<-gsp[,2]; pv<-gsp[,4]; }

ci1<- beta-1.96*se; ci2<- beta+1.96*se

pvx<-substr(rep("****",length(pv)),1,(pv<=0.05)+(pv<=0.01)+(pv<=0.001))

if (colprn==3) {pvv<-pvx;} else {pvv<-pvformat(pv,dec+2);}

if ((colprn!=2) & (gs$family[[2]]=="log" | gs$family[[2]]=="logit")) {

o1<-paste(numfmt(exp(beta),dec)," (",numfmt(exp(ci1),dec),", ",numfmt(exp(ci2),dec),")",sep="")

} else {

if (colprn<3) {o1<-paste(numfmt(beta,dec), " (",numfmt(ci1,dec),", ",numfmt(ci2,dec),")",sep="")

} else {o1<-paste(numfmt(beta,dec), "+",numfmt(se,dec),sep="");}

}

o1<-paste(o1,pvv); o1[is.na(beta)]<-NA

if (length(xxname)>1) {

if (gs$family[[2]]=="log" | gs$family[[2]]=="logit") {

o1[is.na(o1) & substr(xxname,1,7)=="factor("]<-"1.0"

} else {o1[is.na(o1) & substr(xxname,1,7)=="factor("]<-"0";}

o1[is.na(o1)]<-"";

}

return(list(o1,tmpn))

}

recodevar <- function (var,oldcode,newcode) {

tmp.v <- var

nc.tmp <- length(oldcode)

for (i in (1:nc.tmp)) {tmp.v[(var==oldcode[i])]=newcode[i]}

if (is.factor(tmp.v)) {tmp.v1<-as.numeric(as.character(tmp.v))} else {tmp.v1<-as.numeric(tmp.v)}

rm(tmp.v); return(tmp.v1)

}

rankvar <- function(var, num) {

qprobs <- 1/num

if (num>2) {for (i in (2:(num-1))) {qprobs <- c(qprobs, 1/num * i) } }

outvar <- rep(0, times=length(var))

outvar[is.na(var)] <- NA

cutpoints <- quantile(var,probs=qprobs, na.rm=TRUE)

for (k in (1:length(cutpoints))) { outvar[var>=cutpoints[k]] <- k; }

return(outvar)

}

removeNA<-function(i,j,m,wdf) {

vvv<-c(yvname[i],adjvv[[m]],subjvname,colvname,bvar,vname.start,vname.stop,timevname);

if (j<=nx) {vvv<-c(vvv,xvname[j]);} else {vvv<-c(vvv,xvname);}

vvv<-vvv[!is.na(vvv)]; vvv<-vvv[vvv>" "]

tmp<-is.na(wdf[,vvv]);

return(wdf[apply(tmp,1,sum)==0,])

}

vlabelN<-(substr(vlabel,1,1)==" ");

vlabelZ<-vlabel[vlabelN];vlabelV<-vlabel[!vlabelN]

vnameV<-vname[!vlabelN];vnameZ<-vname[vlabelN]

w<-c("<html><head>","<meta http-equiv=\"Content-Type\" content=\"text/html\" charset=\"gb2312\" /></head><body>")

if (!is.na(avname[1])) {

if (sum((saf=="s" | saf=="S") & alv>0)>0) w<-c(w,"</br>Spline smoothing only applies for continuous variables")

if (!is.na(subjvname) & (sum((saf=="s" | saf=="S") & alv==0)>0)) w<-c(w,"</br>Generalized estimate equation could not be used with spline smoothing terms")

}

if (!is.na(svname[1])) {

if (sum((sdf=="s" | sdf=="S") & slv>0)>0) w<-c(w,"</br>Spline smoothing only applies for continuous variables")

if (!is.na(subjvname) & (sum((sdf=="s" | sdf=="S") & slv==0)>0)) w<-c(w,"</br>Generalized estimate equation could not be used with spline smoothing terms")

}

allvname<-c(yvname,xvname,colvname,bvar,avname,svname,subjvname,vname.start,vname.stop,timevname,"weights");

allvname<-allvname[!is.na(allvname)]

WD<-WD[,allvname];

if (!is.na(subjvname)) WD<-WD[order(WD[,subjvname]),]

if (!is.na(sxf[1])) {

if (sum(sxf>1 & xlv>0)>0) w<-c(w,"Categorizing only applies to continuous variables");

if (sum(sxf>1 & xlv==0)>0) {

t.xname<-NA;t.xlv<-NA; nx<-length(xvname)

for (i in 1:nx) {

if (sxf[i]>1 & xlv[i]==0) {

tmp.Xi<- rankvar(WD[,xvname[i]],sxf[i])

tmp.newcode <- tapply(WD[,xvname[i]],tmp.Xi,function(z) median(z,na.rm=TRUE))

tmp.low <- tapply(WD[,xvname[i]],tmp.Xi,function(z) min(z,na.rm=TRUE))

tmp.upp <- tapply(WD[,xvname[i]],tmp.Xi,function(z) max(z,na.rm=TRUE))

tmp.Xi2<- recodevar(tmp.Xi,(1:sxf[i])-1,tmp.newcode)

tmp.Xi<-cbind(tmp.Xi,tmp.Xi2)

tmp.NM<-paste(xvname[i],c("grp","grp.cont"),sep=".")

colnames(tmp.Xi)<-tmp.NM

WD<-cbind(WD,tmp.Xi)

t.xname<-c(t.xname,tmp.NM)

t.xlv<-c(t.xlv,sxf[i],0)

vnameV<-c(vnameV,tmp.NM)

vlabelV<-c(vlabelV,paste(vlabelV[vnameV==xvname[i]],c("group","group trend")))

vnameZ<-c(vnameZ,paste(tmp.NM[1],(1:sxf[i])-1,sep="."))

vlabelZ<-c(vlabelZ,paste(tmp.low,"-",tmp.upp))

} else {

t.xname<-c(t.xname,xvname[i]); t.xlv<-c(t.xlv,xlv[i])

}

}

xvname<-t.xname[-1]; xlv<-t.xlv[-1];

}

}

rm(xv,yv,bv,av,sv,colv,v.start,v.stop)

if (!is.na(subjvname)) {

if (!is.na(avname[1])) saf<-rep(0,length(saf));

if (!is.na(svname[1])) sdf<-rep(0,length(sdf));

WD<-WD[order(WD[,subjvname]),];

}

fmlm<-" "; fmlb<-"Non-adjusted"; tmp<-""; adjvv<-list(NA); adjvb<-"None";

fmlp<-ifelse(!is.na(subjvname), "gee", "glm");

na=0; avb=""; smoothav<-0; nadjm<-0

if (!is.na(avname[1])) {

na<-length(avname)

avb<-vlabelV[match(avname,vnameV)];

avname_ <- avname

smoothavi<-((saf=="s" | saf=="S") & alv==0)

smoothav<-sum(smoothavi)

smoothavname<-avname[smoothavi]

avname_[smoothavi]<-paste("s(",avname[smoothavi],")",sep="")

avb1<-avb

avb1[smoothavi]<-paste(avb[smoothavi],"(Smooth)",sep="")

avname_[alv>0]<-paste("factor(",avname[alv>0],")",sep="")

fmlm<-c(fmlm,paste("+",paste(avname_,collapse="+")))

fmlb<-c(fmlb,"Adjust")

nadjm<-nadjm+1; tmp<-c(tmp,"I"); adjvv[[nadjm+1]]<-avname;

adjvb<-c(adjvb, paste(avb1, collapse="; "))

fmlp<-c(fmlp,ifelse(!is.na(subjvname), "gee", ifelse(smoothav>0, "gam", "glm")))

}

ns=0; svb=""; smoothsv<-0

if (!is.na(svname[1])) {

svb<-vlabelV[match(svname,vnameV)];

svname_ <- svname

smoothsvi<-((sdf=="s" | sdf=="S") & slv==0)

smoothsv<-sum(smoothsvi)

smoothsvname<-svname[smoothsvi]

svname_[smoothsvi]<-paste("s(",svname[smoothsvi],")",sep="")

svb1<-svb

svb1[smoothsvi]<-paste(svb[smoothsvi],"(Smooth)",sep="")

svname_[slv>0]<-paste("factor(",svname[slv>0],")",sep="")

fmlm<-c(fmlm,paste("+",paste(svname_,collapse="+")))

fmlb<-c(fmlb,"Adjust")

nadjm<-nadjm+1; tmp<-c(tmp,"II"); adjvv[[nadjm+1]]<-svname

adjvb<-c(adjvb, paste(svb1, collapse="; "))

fmlp<-c(fmlp,ifelse(!is.na(subjvname), "gee", ifelse(smoothsv>0, "gam", "glm")))

}

if (is.na(parm[1]) & length(fmlm)>1) {

fmlm<-fmlm[-1]; fmlb<-fmlb[-1]; tmp<-tmp[-1]; adjvv<-adjvv[-1]; adjvb<-adjvb[-1]; fmlp<-fmlp[-1];

}

if (nadjm>1) fmlb<-paste(fmlb,tmp)

nmdl<-length(fmlm)

ny=length(yvname); nx=length(xvname);

xb<-vlabelV[match(xvname,vnameV)]; xb[is.na(xb)]<-xvname[is.na(xb)]

yb<-vlabelV[match(yvname,vnameV)]; yb[is.na(yb)]<-yvname[is.na(yb)]

xvname_ <- xvname

xvname_[xlv>0]<-paste("factor(",xvname[xlv>0],")",sep="")

xxname_<-list(NA); xxlbl_<-list(NA); xxlvl_<-list(NA)

for (j in (1:nx)) {

if (xlv[j]==0) {

xxname_[[j+1]]<-xvname[j];xxlbl_[[j+1]]<-xb[j];xxlvl_[[j+1]]<-0

} else {

xxlvl_[[j+1]]<-levels(factor(WD[,xvname[j]]))

tmp<-paste(xvname[j],".",xxlvl_[[j+1]],sep="")

xxlbl_[[j+1]]<-c(xb[j],vlabelZ[match(tmp,vnameZ)])

xxlbl_[[j+1]]<-paste(c("",rep("&nbsp&nbsp",length(xxlbl_[[j+1]])-1)),xxlbl_[[j+1]])

xxname_[[j+1]]<-c(xvname[j],paste("factor(",xvname[j],")",xxlvl_[[j+1]],sep=""))

}

}

xxname_<-xxname_[-1]; xxlbl_<-xxlbl_[-1]; xxlvl_<-xxlvl_[-1];

if (nx==1) par1<-1;

if (is.na(par1)) par1<-1;

if (par1>1) {

tmp1<-xxname_[[1]]; tmp2<-xxlbl_[[1]]

for (j in 2:nx) {tmp1<-c(tmp1,xxname_[[j]]); tmp2<-c(tmp2,xxlbl_[[j]]);}

xxname_[[nx+1]]<-tmp1; xxlbl_[[nx+1]]<-tmp2;

xvname_<-c(xvname_,paste(xvname_,collapse="+"))

}

contx<-(sum(xlv>0)==0)

if (par1==3 & !is.na(bvar)) {w<-c(w,"</br>Column stratified variable was ignored"); bvar<-NA; bvname<-NA;}

if (is.na(bvar) & !is.na(colvname) & nmdl==1 & par1!=3) {if ((ny==1) | (nx==1 & contx)) {bvar<-colvname; colvname<-NA;}}

if (is.na(colvname)) {

nclv<-1; clvb<-"Total"; clvb_<-"Total"

} else {

clv<-levels(factor(WD[,colvname])); nclv<-length(clv)+1

clvb_<-vlabelZ[match(paste(colvname,".",clv,sep=""),vnameZ)]; clvb_[is.na(clvb_)]<-clv[is.na(clvb_)];

clvb<-c(paste(vlabelV[vnameV==colvname],clvb_,sep="="),"Total");

clvb_<-c(clvb_,"Total")

WD<-WD[!is.na(WD[,colvname]),]

}

if (is.na(bvar)) {

blvb<-"Total"; blvb_<-"Total"

} else {

blv<-levels(factor(WD[,bvar])); nblv<-length(blv)+1

blvb_<-vlabelZ[match(paste(bvar,".",blv,sep=""),vnameZ)]; blvb_[is.na(blvb_)]<-blv[is.na(blvb_)];

blvb<-c(paste(vlabelV[vnameV==bvar],blvb_,sep="="),"Total");

blvb_<-c(blvb_,"Total")

WD<-WD[!is.na(WD[,bvar]),]

}

aa<-c(1,2,3,4)

for (i in 1:4) {

for (j in c(1:4)[-i]) {

for (k in c(1:4)[-c(i,j)]) aa<-rbind(aa,c(i,j,k,c(1:4)[-c(i,j,k)]))

}

}

if (is.na(parm[4])) parm[4]<-1

rord<-aa[parm[4],]

if (!is.na(bvar)) {prn<-"S";

} else {

if (parm[4]>1) {

rordc<-ifelse(rord[4]==1,3,4)

prn<-c("G","Y","M","X")[rord[rordc]]

if (prn=="X") {

if (!contx & nx>1) prn<-ifelse(nmdl>1, "M", ifelse(ny>nx & contx, "X", "Y"))

if (!contx & nx==1) prn<-"CX"

if (par1==2) {tmp<-ifelse(rordc==4,ifelse(rord[3]==1,2,3),2); prn<-c("G","Y","M","X")[rord[tmp]];}

}

} else {

prn<-ifelse(nmdl>1, "M", ifelse(ny>nx & contx, "X", "Y"))

if (par1==2) prn<-ifelse(nmdl>1, "M", "Y")

}

if (par1==3) prn<-"UM"

}

colprn<-parm[3];

sink(paste(ofname,".lst",sep=""))

if (par1==2) {xbgn<-nx+1; xend<-nx+1;} else {xbgn<-1; xend<-nx;}

if (prn=="Y") {

tt<-c(0,0,0,0,"Exposure",yb); nn<-c(0,0,0,0,yb);

for (k in (1:nclv)) {

wdtmp0<-WD;

if (!is.na(colvname)) {

if (k<nclv) wdtmp0<-WD[WD[,colvname]==clv[k],];

print(paste("Stratified by",colvname, ":", clvb[k]))

}

for (m in 1:nmdl) {

for (j in (xbgn:xend)) {

colj<-cbind(k,0,m,j,xxlbl_[[j]])

nnj <-c(k,0,m,j)

for (i in (1:ny)) {

fml<-paste(yvname[i],"~",xvname_[j],fmlm[m]);

wdtmp<-removeNA(i,j,m,wdtmp0)

if (!is.na(colvname)) {if (k==nclv) fml<-paste(fml,"+factor(",colvname,")",sep="");}

if (fmlp[m]=="gam") tmp.mdl<-setgam(fml,i)

if (fmlp[m]=="gee") tmp.mdl<-setgee(fml,i)

if (fmlp[m]=="glm") tmp.mdl<-setglm(fml,i)

tmpooi<-mdl2oo(tmp.mdl,xxname_[[j]],fmlp[m])

colj<-cbind(colj,tmpooi[[1]]); nnj<-c(nnj,tmpooi[[2]])

}

tt<-rbind(tt,colj); nn<-rbind(nn,nnj)

}

}

}

}

if (prn=="S") {

tt<-c(0,0,0,0,"Exposure",blvb); nn<-c(0,0,0,0,blvb);

for (k in (1:nclv)) {

wdtmp0<-WD;

if (!is.na(colvname)) {

if (k<nclv) wdtmp0<-WD[WD[,colvname]==clv[k],];

print(paste("Stratified by",colvname, ":", clvb[k]))

}

for (i in (1:ny)) {

for (m in 1:nmdl) {

for (j in (xbgn:xend)) {

colj<-cbind(k,i,m,j,xxlbl_[[j]]);

nnj <- c(k,i,m,j)

for (b in (1:nblv)) {

print(paste("Stratified by",bvar, ":", blvb[b]))

fml<-paste(yvname[i],"~",xvname_[j],fmlm[m]);

if (b<nblv) {

wdtmp1<-wdtmp0[wdtmp0[,bvar]==blv[b],];

} else {

wdtmp1<-wdtmp0; fml<-paste(fml,"+factor(",bvar,")",sep="");

}

wdtmp<-removeNA(i,j,m,wdtmp1)

if (!is.na(colvname)) {if (k==nclv) fml<-paste(fml,"+factor(",colvname,")",sep="");}

if (fmlp[m]=="gam") tmp.mdl<-setgam(fml,i)

if (fmlp[m]=="gee") tmp.mdl<-setgee(fml,i)

if (fmlp[m]=="glm") tmp.mdl<-setglm(fml,i)

tmpooi<-mdl2oo(tmp.mdl,xxname_[[j]],fmlp[m])

colj<-cbind(colj,tmpooi[[1]]); nnj<-c(nnj,tmpooi[[2]])

}

tt<-rbind(tt,colj); nn<-rbind(nn,nnj)

}

}

}

}

}

if (prn=="M") {

tt<-c(0,0,0,0,"Exposure",fmlb); nn<-c(0,0,0,0,fmlb)

for (k in (1:nclv)) {

wdtmp0<-WD;

if (!is.na(colvname)) {

if (k<nclv) wdtmp0<-WD[WD[,colvname]==clv[k],];

print(paste("Stratified by",colvname, ":", clvb[k]))

}

for (i in 1:ny) {

for (j in xbgn:xend) {

colj<-cbind(k,i,0,j,xxlbl_[[j]]); nnj<-c(k,i,0,j)

for (m in 1:nmdl) {

fml<-paste(yvname[i],"~",xvname_[j],fmlm[m]);

wdtmp<-removeNA(i,j,m,wdtmp0)

if (!is.na(colvname)) {if (k==nclv) fml<-paste(fml,"+factor(",colvname,")",sep="");}

if (fmlp[m]=="gam") tmp.mdl<-setgam(fml,i)

if (fmlp[m]=="gee") tmp.mdl<-setgee(fml,i)

if (fmlp[m]=="glm") tmp.mdl<-setglm(fml,i)

tmpooi<-mdl2oo(tmp.mdl,xxname_[[j]],fmlp[m])

colj<-cbind(colj,tmpooi[[1]]); nnj<-c(nnj,tmpooi[[2]])

}

tt<-rbind(tt,colj); nn<-rbind(nn,nnj)

}

}

}

}

if (prn=="X") {

tt<-c(0,0,0,0,"Outcome",xb); nn<-c(0,0,0,0,xb);

for (k in (1:nclv)) {

wdtmp0<-WD;

if (!is.na(colvname)) {

if (k<nclv) wdtmp0<-WD[WD[,colvname]==clv[k],];

print(paste("Stratified by",colvname, ":", clvb[k]))

}

for (m in 1:nmdl) {

for (i in (1:ny)) {

colj<-cbind(k,i,m,0,yb[i])

nnj <-c(k,i,m,0)

for (j in (1:nx)) {

fml<-paste(yvname[i],"~",xvname_[j],fmlm[m]);

wdtmp<-removeNA(i,j,m,wdtmp0)

if (!is.na(colvname)) {if (k==nclv) fml<-paste(fml,"+factor(",colvname,")",sep="");}

if (fmlp[m]=="gam") tmp.mdl<-setgam(fml,i)

if (fmlp[m]=="gee") tmp.mdl<-setgee(fml,i)

if (fmlp[m]=="glm") tmp.mdl<-setglm(fml,i)

tmpooi<-mdl2oo(tmp.mdl,xxname_[[j]],fmlp[m])

colj<-cbind(colj,tmpooi[[1]]); nnj<-c(nnj,tmpooi[[2]])

}

tt<-rbind(tt,colj); nn<-rbind(nn,nnj)

}

}

}

}

if (prn=="CX") {

tt<-c(0,0,0,0,"Outcome",xxlbl_[[1]][-1]); nn<-c(0,0,0,0,xb[1]);

nxl<-length(xxlbl_[[1]])-1

for (k in (1:nclv)) {

wdtmp0<-WD;

if (!is.na(colvname)) {

if (k<nclv) wdtmp0<-WD[WD[,colvname]==clv[k],];

print(paste("Stratified by",colvname, ":", clvb[k]))

}

for (m in 1:nmdl) {

for (i in (1:ny)) {

colj<-c(k,i,m,0,yb[i])

nnj <-c(k,i,m,0)

fml<-paste(yvname[i],"~",xvname_[1],fmlm[m]);

wdtmp<-removeNA(i,1,m,wdtmp0)

if (!is.na(colvname)) {if (k==nclv) fml<-paste(fml,"+factor(",colvname,")",sep="");}

if (fmlp[m]=="gam") tmp.mdl<-setgam(fml,i)

if (fmlp[m]=="gee") tmp.mdl<-setgee(fml,i)

if (fmlp[m]=="glm") tmp.mdl<-setglm(fml,i)

tmpooi<-mdl2oo(tmp.mdl,xxname_[[1]],fmlp[m])

colj<-c(colj,tmpooi[[1]][-1]); nnj<-c(nnj,tmpooi[[2]])

tt<-rbind(tt,colj); nn<-rbind(nn,nnj)

}

}

}

}

if (prn=="UM") {

tt<-c(0,0,0,0,"Exposure","Univariable","Multivariable"); nn<-c(0,0,0,0,"Univariable","Multivariable")

for (k in (1:nclv)) {

wdtmp0<-WD;

if (!is.na(colvname)) {

if (k<nclv) wdtmp0<-WD[WD[,colvname]==clv[k],];

print(paste("Stratified by",colvname, ":", clvb[k]))

}

for (i in 1:ny) {

for (m in 1:nmdl) {

colm<-rep(NA,6); nnm<-rep(NA,5)

for (j in 1:(nx+1)) {

colj<-cbind(k,i,m,j,xxlbl_[[j]]); nnj<-c(k,i,m,j)

fml<-paste(yvname[i],"~",xvname_[j],fmlm[m]);

wdtmp<-removeNA(i,j,m,wdtmp0)

if (!is.na(colvname)) {if (k==nclv) fml<-paste(fml,"+factor(",colvname,")",sep="");}

if (fmlp[m]=="gam") tmp.mdl<-setgam(fml,i)

if (fmlp[m]=="gee") tmp.mdl<-setgee(fml,i)

if (fmlp[m]=="glm") tmp.mdl<-setglm(fml,i)

tmpooi<-mdl2oo(tmp.mdl,xxname_[[j]],fmlp[m])

colj<-cbind(colj,tmpooi[[1]]); nnj<-c(nnj,tmpooi[[2]])

if (j<=nx) {colm<-rbind(colm,colj); nnm<-rbind(nnm,nnj);

} else {colm<-cbind(colm[-1,],tmpooi[[1]]); nnm<-cbind(nnm[-1,],tmpooi[[2]]);}

}

tt<-rbind(tt,colm); nn<-rbind(nn,nnm)

}

}

}

}

sink()

if (!contx & prn!="CX") rord<-c(rord[rord!=4],4)

if (prn=="X" | prn=="CX") rord<-rord[rord!=4]

if (prn=="Y") rord<-rord[rord!=2]

if (prn=="M") rord<-rord[rord!=3]

if (nx==1 & contx) rord<-rord[rord!=4]

if (nmdl==1) rord<-rord[rord!=3]

if (ny==1) rord<-rord[rord!=2]

if (is.na(colvname)) rord<-rord[rord!=1]

if (length(rord)==0) rord<-1

nrr<-length(rord)

for (i in nrr:1) {nn<-nn[order(as.numeric(nn[,rord[i]])),];tt<-tt[order(as.numeric(tt[,rord[i]])),];}

if (nn[2,4]>0) {nn[,4]<-c("Exposure",xb[as.numeric(nn[-1,4])]); } else {nn<-nn[,-4];}

if (nn[2,3]>0) {nn[,3]<-c("Model",fmlb[as.numeric(nn[-1,3])]); } else {nn<-nn[,-3];}

if (nn[2,2]>0) {nn[,2]<-c("Outcome",yb[as.numeric(nn[-1,2])]); } else {nn<-nn[,-2];}

if (!is.na(colvname)) {nn[,1]<-c(vlabel[vname==colvname],clvb_[as.numeric(nn[-1,1])]);} else {nn<-nn[,-1];}

tb<-matrix(as.numeric(tt[,c(1:4)]),ncol=4);

if (!is.na(colvname)) {tt[,1]<-c(vlabelV[vnameV==colvname],clvb[tb[-1,1]]);}

if (ny>1) {tt[,2]<-c("Outcome",yb[tb[-1,2]]);}

if (nmdl>1) {tt[,3]<-c("Model",fmlb[tb[-1,3]]);}

nrr1<-nrr-1; oo<-tt[1,]; nc<-ncol(tt)-5; nr<-nrow(tt)

for (i in 2:nr) {

if (nrr>1) {

for (j in 1:nrr1) {

if (tb[i,rord[j]]!=tb[i-1,rord[j]]) oo<-rbind(oo,c(rep(tt[i,rord[j]],5),rep(" ",nc)))

}

}

oo<-rbind(oo,tt[i,])

}

if (rord[nrr]!=4 & tt[1,rord[nrr]]!="0") {oo<-cbind(oo[,rord[nrr]],oo[,-(1:5)]);} else {oo<-oo[,-(1:4)]}

w<-c(w,paste("<h2>", title, "</h2>"))

w<-c(w,"</br><table border=3>", mat2htmltable(oo), "</table>")

prnopt<-c("β (95%CI) Pvalue / OR (95%CI) Pvalue", "β (95%CI) Pvalue", "β+se / OR (95%CI) *P<0.05 **P<0.01 ***P<0.001")

library(mgcv,lib.loc=R.LibLocation)

library(gdata,lib.loc=R.LibLocation)

ofname<-"PROJ11_5_tbl";

WD<-EmpowerStatsR; wd.subset="";

svy.DSN.YN <- FALSE;

weights<-WD$WTINT2YR;weights.var <- 'wtint2yr';

WD<-cbind(WD,weights); WD<-WD[!is.na(weights),];

title<-"Figure 2";

attach(WD)

subjvname<-NA;

yv<-cbind(BMD);

yvname<-c('BMD');

yvar<-c('BMD');

ydist<-c('gaussian');

ylink<-c('identity');

ylv<-c(0);

xv<-cbind(ALQ101,BP,BMI,HBAIC,GENE,AGE,RACE,DMDEDUC1,DIQ,ALBUMIN,UREA,CA,TC,CREATININE,P,TG);

xvname<-c('ALQ101','BP','BMI','HBAIC','GENE','AGE','RACE','DMDEDUC1','DIQ','ALBUMIN','UREA','CA','TC','CREATININE','P','TG');

xvar<-c('ALQ101','BP','BMI','HBAIC','GENE','AGE','RACE','DMDEDUC1','DIQ','ALBUMIN','UREA','CA','TC','CREATININE','P','TG');

xlv<-c(3,3,0,0,2,0,4,4,3,0,0,0,0,0,0,0);

sxf<-c(NA,0,0,0,0,0,0,0,0,0,0,0,0,0,0,0,0)[-1];

sv<-cbind(PLR,NLR);

svname<-c('PLR','NLR');

svar<-c('PLR','NLR');

sdf<-c(NA,0,0)[-1];

slv<-c(0,0);

av<-NA; avname<-NA; avlbl<-NA; nadj<-0; alv<-NA;

timev<-NA; timevname<-NA;

bv<-NA; bvar<-NA;

colv<-NA; colvname<-NA;

v.start<-NA; vname.start<-NA;

v.stop<-NA; vname.stop<-NA;

par1<-1;dec<-4;parm<-c(NA, NA, NA, NA, 0);

if (!exists("pdfwd")) pdfwd<-6;

if (!exists("pdfht")) pdfht<-6;

##R package## mgcv gdata ##R package##;

vec2shift<-function(vnew,vorg,f, opt) {

if (is.na(f[1])) {

mean1<-mean(vorg)

if (opt=="logit") mean1<-log(mean1/(1-mean1))

if (opt=="log") mean1<-log(mean1)

vnew<-vnew+(mean1-mean(vnew))

} else {

mean1<-tapply(vorg,factor(f),mean)

if (opt=="logit") mean1<-log(mean1/(1-mean1))

if (opt=="log") mean1<-log(mean1)

mean2<-tapply(vnew,factor(f),mean); meand<-mean1-mean2; lvf<-levels(factor(f))

for (z in (1:length(lvf))) {vnew[factor(f)==lvf[z]]<-vnew[factor(f)==lvf[z]]+meand[z]; }

}

return(vnew)

}

getNumber<-function(str, n) {

str<-substr(str,2,nchar(str)-1)

for (i in (1:nchar(str))) {if (substr(str,i,i)==",") {p=i; break}; }

ifelse(n==1,return(substr(str,1,p-1)),return(substr(str,p+1,nchar(str))))

}

legLocate<-function(x,y) {

x[is.infinite(y)]<-NA

y[is.infinite(y)]<-NA

xmin<-min(x,na.rm=TRUE); xmax<-max(x,na.rm=TRUE)

ymin<-min(y,na.rm=TRUE); ymax<-max(y,na.rm=TRUE)

yoff<-(ymax-ymin); tmp<-table(cut(x,3),cut(y,4))

tmp.r=which.min(tmp[,4]);tmp.c=4

if (tmp[2,1]==0) {tmp.r=2;tmp.c=1}

if (tmp[1,1]==0) {tmp.r=1;tmp.c=1}

if (tmp[3,1]==0) {tmp.r=3;tmp.c=1}

if (tmp[2,4]==0) {tmp.r=2;tmp.c=4}

if (tmp[1,4]==0) {tmp.r=1;tmp.c=4}

if (tmp[3,4]==0) {tmp.r=3;tmp.c=4}

pos.y<-colnames(tmp)[tmp.c]; pos.x<-rownames(tmp)[tmp.r]; pct<-0.15

if (tmp.c==4) {

if (min(tmp[,4])>0) {pct<-0.3}

ymax<-ymax+yoff*pct; legy<-ymax;ymin<-ymin-yoff*0.1

}

if (tmp.c==1) {

if (min(tmp[,1])>0) {pct<-0.3}

legy<-as.numeric(getNumber(pos.y,2));ymin<-ymin-yoff*pct;ymax=ymax+yoff*0.1

}

legx<-as.numeric(getNumber(pos.x,1))

return(cbind(xmin,xmax,ymin,ymax,legx,legy))

}

mat2htmltable<-function(mat) {

t1<- apply(mat,1,function(z) paste(z,collapse="</td><td>"))

t2<- paste("<tr><td>",t1,"</td></tr>")

return(paste(t2,collapse=" "))

}

setgam<-function(fml,yi) {

if (ydist[yi]=="") ydist[yi]<-"gaussian"

if (ydist[yi]=="exact") ydist[yi]<-"binomial"

if (ydist[yi]=="breslow") ydist[yi]<-"binomial"

if (ydist[yi]=="gaussian") mdl<-gam(formula(fml),weights=wd$weights,data=wd, family=gaussian(link="identity"))

if (ydist[yi]=="binomial") mdl<-gam(formula(fml),weights=wd$weights,data=wd, family=binomial(link="logit"))

if (ydist[yi]=="poisson") mdl<-gam(formula(fml),weights=wd$weights,data=wd, family=poisson(link="log"))

if (ydist[yi]=="gamma") mdl<-gam(formula(fml),weights=wd$weights,data=wd, family=Gamma(link="inverse"))

if (ydist[yi]=="negbin") mdl<-gam(formula(fml),weights=wd$weights,data=wd, family=negbin(c(1,10), link="log"))

return(mdl)

}

gam2htmltable<-function(mdl) {

gs<-summary(mdl)

np<-length(gs$p.coeff)

coe<-gs$p.table

if (gs$family[[2]]=="log" | gs$family[[2]]=="logit") {

cnames<-c(colnames(coe),"exp(est)","95%CI low","95%CI upp")

coe<- cbind(coe, exp(coe[,1]), exp(coe[,1]-1.96*coe[,2]), exp(coe[,1]+1.96*coe[,2]))

}

if (gs$family[[2]]=="identity") {

cnames<-c(colnames(coe),"95%CI low","95%CI upp")

coe<- cbind(coe, coe[,1]-1.96*coe[,2], coe[,1]+1.96*coe[,2])

}

oo1<-cbind(c("",rownames(coe)),rbind(cnames,round(coe,dec)))

oo<-c("</br>Linear terms effect<table border=3>",mat2htmltable(oo1),"</table>")

if (!is.null(gs$pTerms.table)) {

xsq<-gs$pTerms.table

oo2<-cbind(c("",rownames(xsq)),rbind(colnames(xsq),round(xsq,dec)))

oo<-c(oo, "</br>Chi-square tests for linear terms<table border=3>",mat2htmltable(oo2),"</table>")

}

if (!is.null(gs$s.table)) {

stb<-gs$s.table

oo3<-cbind(c("",rownames(stb)),rbind(colnames(stb),round(stb,dec)))

oo<-c(oo, "</br>Approximate significance of smooth terms<table border=3>",mat2htmltable(oo3),"</table>")

}

p0<-c("N:", gs$n)

p1<-c("Adj. r-square:", round(gs$r.sq,4))

p2<-c("Deviance explained:", round(gs$dev.expl,4))

p3<-c("UBRE score (sp.criterion):", round(gs$sp.criterion,4))

p4<-c("Scale estimate:", gs$scale)

p5<-c("family:", gs$family[[1]])

p6<-c("link function:", gs$family[[2]])

oo4<-rbind(p0,p1,p2,p3,p4,p5,p6)

oo<-c(oo, "</br>Model statistics<table border=3>",mat2htmltable(oo4),"</table>")

return(oo)

}

gam2pngs<-function(mdl,yi,xi) {

pred<-predict.gam(mdl,type="terms",se.fit=TRUE)

mfit<-NA; sfit<-NA; tmp.cname<-NA; kk0<-NA

if (xi==0) {kb=1; ke=ns;} else {kb=xi; ke=xi;}

for (k in (kb:ke)) {

if (slv[k]==0) {

mfit<-cbind(mfit,apply(cbind(0,pred$fit[,sxterms[k,]]),1,sum));

sfit<-cbind(sfit,apply(cbind(0,pred$se.fit[,sxterms[k,]]),1,sum));

tmp.cname<-c(tmp.cname,svname[k])

kk0<-c(kk0,k)

}

}

tmp.cname<-tmp.cname[-1]; kk0<-kk0[-1]

mfit<-matrix(mfit[,-1],ncol=length(tmp.cname)); colnames(mfit)<-paste(tmp.cname,".fit",sep="");

sfit<-matrix(sfit[,-1],ncol=length(tmp.cname)); colnames(sfit)<-paste(tmp.cname,".se",sep="");

if (!is.na(colvname)) {tmpfac<-wd[,colvname];} else {tmpfac<-NA;}

if (mdl$family[2]=="logit") {

mfit<-apply(mfit,2,function(z) vec2shift(z,mdl$fitted.value,tmpfac,"logit"))

mfit.low<-mfit-1.96*sfit; mfit.low<-matrix(exp(mfit.low)/(1+exp(mfit.low)),ncol=length(tmp.cname))

mfit.upp<-mfit+1.96*sfit; mfit.upp<-matrix(exp(mfit.upp)/(1+exp(mfit.upp)),ncol=length(tmp.cname))

mfit<-matrix(exp(mfit)/(1+exp(mfit)),ncol=length(tmp.cname))

colnames(mfit.low)<-paste(tmp.cname,".low",sep="");

colnames(mfit.upp)<-paste(tmp.cname,".upp",sep="");

colnames(mfit)<-paste(tmp.cname,".fit",sep="");

ww<-cbind(wd,mfit); if (is.na(colvname)) ww<-cbind(ww,mfit.low,mfit.upp)

} else if (mdl$family[2]=="log") {

mfit<-apply(mfit,2,function(z) vec2shift(z,mdl$fitted.value,tmpfac,"log"))

mfit.low<-mfit-1.96*sfit; mfit.low<-matrix(exp(mfit.low),ncol=length(tmp.cname))

mfit.upp<-mfit+1.96*sfit; mfit.upp<-matrix(exp(mfit.upp),ncol=length(tmp.cname))

mfit<-matrix(exp(mfit),ncol=length(tmp.cname))

colnames(mfit.low)<-paste(tmp.cname,".low",sep="");

colnames(mfit.upp)<-paste(tmp.cname,".upp",sep="");

colnames(mfit)<-paste(tmp.cname,".fit",sep="");

ww<-cbind(wd,mfit); if (is.na(colvname)) ww<-cbind(ww,mfit.low,mfit.upp)

} else if (mdl$family[2]=="identity") {

mfit<-apply(mfit,2,function(z) vec2shift(z,mdl$fitted.value,tmpfac," "))

ww<-cbind(wd,mfit,sfit)

} else {

ww<-cbind(wd,mfit,sfit)

}

if (xi!=0) {xf<-paste(ofname,yvar[yi],svar[xi],"gam.xls",sep="_");} else {xf<-paste(ofname,yvar[yi], "gam.xls", sep="_");}

write.table(ww,file=xf,row.names=FALSE,col.names=TRUE,sep="\t",append=FALSE,quote=FALSE)

px<-c(20,1:9); gg<-"";

for (k in kk0) {

cname1<-paste(svname[k],".fit",sep=""); y.tmp<-mfit[,cname1]

if (mdl$family[2]=="logit" | mdl$family[2]=="log") {

cname2<-paste(svname[k],".low",sep=""); y.low<-mfit.low[,cname2]

cname3<-paste(svname[k],".upp",sep=""); y.upp<-mfit.upp[,cname3]

} else {

cname2<-paste(svname[k],".se",sep=""); se.tmp<-sfit[,cname2];

y.low<-y.tmp-1.96*se.tmp; y.upp<-y.tmp+1.96*se.tmp

}

x.tmp<-wd[,svname[k]];

pngf<-paste(ofname,yvar[yi],svar[k],"smooth.png",sep="_")

pdff<-paste(ofname,yvar[yi],svar[k],"smooth.pdf",sep="_")

pngf0<-paste(ofname,yvar[yi],svar[k],"smooth1.png",sep="_")

pdff0<-paste(ofname,yvar[yi],svar[k],"smooth1.pdf",sep="_")

if (is.na(colvname)) {

if (is.na(parm[1])) {tmp.col<-c("red","blue");} else {tmp.col<-rep("black",2);}

xy<-legLocate(c(x.tmp,x.tmp),c(y.low,y.upp))

png(pngf,width=720,height=560)

plot(y.tmp~x.tmp,ylim=c(xy[3],xy[4]),xlim=c(xy[1],xy[2]),col=tmp.col[1],type="p", pch=20, ylab="", xlab="")

par(new=TRUE);

plot(y.low~x.tmp,ylim=c(xy[3],xy[4]),xlim=c(xy[1],xy[2]),col=tmp.col[2], type="p", pch=1, ylab="", xlab="")

par(new=TRUE);

plot(y.upp~x.tmp,ylim=c(xy[3],xy[4]),xlim=c(xy[1],xy[2]),col=tmp.col[2], type="p", pch=1, ylab=yb[yi], xlab=sb[k])

dev.off()

pdf(pdff,width=pdfwd, height=pdfht, family="Helvetica");

plot(y.tmp~x.tmp,ylim=c(xy[3],xy[4]),xlim=c(xy[1],xy[2]),col=tmp.col[1],type="p", pch=20, ylab="", xlab="")

par(new=TRUE);

plot(y.low~x.tmp,ylim=c(xy[3],xy[4]),xlim=c(xy[1],xy[2]),col=tmp.col[2], type="p", pch=1, ylab="", xlab="")

par(new=TRUE);

plot(y.upp~x.tmp,ylim=c(xy[3],xy[4]),xlim=c(xy[1],xy[2]),col=tmp.col[2], type="p", pch=1, ylab=yb[yi], xlab=sb[k])

dev.off()

png(pngf0,width=720,height=560)

tmp.ord<-order(x.tmp); x.tmp0<-x.tmp[tmp.ord];

y.tmp0<-y.tmp[tmp.ord];y.low0<-y.low[tmp.ord];y.upp0<-y.upp[tmp.ord]

plot(y.tmp0~x.tmp0,ylim=c(xy[3],xy[4]),xlim=c(xy[1],xy[2]),col=tmp.col[1],type="l", lty=1, lwd=2, ylab="", xlab="")

par(new=TRUE);

plot(y.low0~x.tmp0,ylim=c(xy[3],xy[4]),xlim=c(xy[1],xy[2]),col=tmp.col[2], type="l", lty=3, lwd=1, ylab="", xlab="")

par(new=TRUE);

plot(y.upp0~x.tmp0,ylim=c(xy[3],xy[4]),xlim=c(xy[1],xy[2]),col=tmp.col[2], type="l", lty=3, lwd=1, ylab=yb[yi], xlab=sb[k])

rug(x.tmp0)

dev.off()

pdf(pdff0,width=pdfwd, height=pdfht, family="Helvetica");

tmp.ord<-order(x.tmp); x.tmp0<-x.tmp[tmp.ord];

y.tmp0<-y.tmp[tmp.ord];y.low0<-y.low[tmp.ord];y.upp0<-y.upp[tmp.ord]

plot(y.tmp0~x.tmp0,ylim=c(xy[3],xy[4]),xlim=c(xy[1],xy[2]),col=tmp.col[1],type="l", lty=1, lwd=2, ylab="", xlab="")

par(new=TRUE);

plot(y.low0~x.tmp0,ylim=c(xy[3],xy[4]),xlim=c(xy[1],xy[2]),col=tmp.col[2], type="l", lty=3, lwd=1, ylab="", xlab="")

par(new=TRUE);

plot(y.upp0~x.tmp0,ylim=c(xy[3],xy[4]),xlim=c(xy[1],xy[2]),col=tmp.col[2], type="l", lty=3, lwd=1, ylab=yb[yi], xlab=sb[k])

rug(x.tmp0)

dev.off()

rm(tmp.ord,y.tmp0,x.tmp0,y.low0,y.upp0)

xy<-legLocate(x.tmp,wd[,1])

pngf1<-paste(ofname,yvar[yi],svar[k],"scatter.png",sep="_")

pdff1<-paste(ofname,yvar[yi],svar[k],"scatter.pdf",sep="_")

png(pngf1,width=720,height=560)

plot(y.tmp~x.tmp,ylim=c(xy[3],xy[4]),xlim=c(xy[1],xy[2]),col=tmp.col[1], type="p", pch=20, ylab="", xlab="")

par(new=TRUE);

plot(wd[,1]~x.tmp,ylim=c(xy[3],xy[4]),xlim=c(xy[1],xy[2]),type="p",pch=1,cex=0.5, ylab=yb[yi], xlab=sb[k])

dev.off()

pdf(pdff1,width=pdfwd, height=pdfht, family="Helvetica");

plot(y.tmp~x.tmp,ylim=c(xy[3],xy[4]),xlim=c(xy[1],xy[2]),col=tmp.col[1], type="p", pch=20, ylab="", xlab="")

par(new=TRUE);

plot(wd[,1]~x.tmp,ylim=c(xy[3],xy[4]),xlim=c(xy[1],xy[2]),type="p",pch=1,cex=0.5, ylab=yb[yi], xlab=sb[k])

dev.off()

} else {

if (is.na(parm[1])) {tmp.col<-rainbow(ncg);tmp.col1<-c("red","blue")} else {tmp.col<-rep("black",ncg);tmp.col1<-c("black","black")}

for (b in (1:ncg)) {

y00<-y.tmp[wd[,colvname]==colv.lv[b]]; x00<-x.tmp[wd[,colvname]==colv.lv[b]]

xy1<-legLocate(x00,y00)

pngf1<-paste(ofname,yvar[yi],svar[k],colvname,colv.lv[b],"smooth.png",sep="_")

pdff1<-paste(ofname,yvar[yi],svar[k],colvname,colv.lv[b],"smooth.pdf",sep="_")

png(pngf1,width=720,height=560)

plot(y00~x00,ylim=c(xy1[3],xy1[4]),xlim=c(xy1[1],xy1[2]),col=tmp.col1[1],type="p", pch=20, ylab=yb[yi], xlab=sb[k])

dev.off()

pdf(pdff1,width=pdfwd, height=pdfht, family="Helvetica");

plot(y00~x00,ylim=c(xy1[3],xy1[4]),xlim=c(xy1[1],xy1[2]),col=tmp.col1[1],type="p", pch=20, ylab=yb[yi], xlab=sb[k])

dev.off()

}

xy<-legLocate(x.tmp,y.tmp)

png(pngf,width=720,height=560)

for (b in (1:ncg)) {

y0<-y.tmp[wd[,colvname]==colv.lv[b]]; x0<-x.tmp[wd[,colvname]==colv.lv[b]]

if (b>1) par(new=TRUE)

plot(y0~x0,ylim=c(xy[3],xy[4]),xlim=c(xy[1],xy[2]),col=tmp.col[b], type="p", pch=px[b], ylab=yb[yi], xlab=sb[k])

}

legend(xy[5],xy[6],colv.lb,title=colvb, pch=px[1:ncg],bty="n",col=tmp.col)

dev.off()

pdf(pdff,width=pdfwd, height=pdfht, family="Helvetica");

for (b in (1:ncg)) {

y0<-y.tmp[wd[,colvname]==colv.lv[b]]; x0<-x.tmp[wd[,colvname]==colv.lv[b]]

if (b>1) par(new=TRUE)

plot(y0~x0,ylim=c(xy[3],xy[4]),xlim=c(xy[1],xy[2]),col=tmp.col[b], type="p", pch=px[b], ylab=yb[yi], xlab=sb[k])

}

legend(xy[5],xy[6],colv.lb,title=colvb, pch=px[1:ncg],bty="n",col=tmp.col)

dev.off()

png(pngf0,width=720,height=560)

for (b in (1:ncg)) {

y0<-y.tmp[wd[,colvname]==colv.lv[b]]; x0<-x.tmp[wd[,colvname]==colv.lv[b]]

tmp.ord<-order(x0); x00<-x0[tmp.ord]; y00<-y0[tmp.ord];

if (b>1) par(new=TRUE)

plot(y00~x00,ylim=c(xy[3],xy[4]),xlim=c(xy[1],xy[2]),col=tmp.col[b], type="l", lty=b, lwd=2, ylab=yb[yi], xlab=sb[k])

rm(tmp.ord,x00,y00)

}

legend(xy[5],xy[6],colv.lb,title=colvb,lty=(1:ncg),bty="n",col=tmp.col)

dev.off()

pdf(pdff0,width=pdfwd, height=pdfht, family="Helvetica");

for (b in (1:ncg)) {

y0<-y.tmp[wd[,colvname]==colv.lv[b]]; x0<-x.tmp[wd[,colvname]==colv.lv[b]]

tmp.ord<-order(x0); x00<-x0[tmp.ord]; y00<-y0[tmp.ord];

if (b>1) par(new=TRUE)

plot(y00~x00,ylim=c(xy[3],xy[4]),xlim=c(xy[1],xy[2]),col=tmp.col[b], type="l", lty=b, lwd=2, ylab=yb[yi], xlab=sb[k])

rm(tmp.ord,x00,y00)

}

legend(xy[5],xy[6],colv.lb,title=colvb,lty=(1:ncg),bty="n",col=tmp.col)

dev.off()

}

gg<-c(gg,"<td>",yb[yi]," vs. ",sb[k],"</br><a href=\"",pngf,"\" target=_BLANK><img src=\"",pngf,"\" width=320,height=320></a></td>")

}

return(gg)

}

adjmean<-function(mdl, yi, xi) {

if (!is.na(xvname[1])) {allvname<- c(xvname,svname); all.lv<-c(xlv,slv);} else {allvname<-c(svname); all.lv<-slv;}

if (!is.na(colvname)) allvname<-c(allvname,colvname)

nv = length(allvname)

xi.lv <- levels(factor(WD[,svname[xi]]))

newd0 <- matrix(0,ncol=nv,nrow=length(xi.lv))

colnames(newd0)<-allvname

for (b in 1:length(all.lv)) {

if (all.lv[b]==0) {

newd0[,b]<-mean(WD[,allvname[b]],na.rm=TRUE)

} else {

uniqv <- unique(WD[,allvname[b]])

newd0[,b]<-uniqv[which.max(tabulate(match(WD[,allvname[b]], uniqv)))]

}

}

newd0[,svname[xi]]<-as.numeric(xi.lv)

if (!is.na(colvname)) {

for (b in 1:ncg) {

newd1<-newd0; newd1[,colvname]<-as.numeric(colv.lv[b]);

if (b==1) {newD<-newd1;} else {newD<-rbind(newD,newd1);}

}

f<-table(wd[,svname[xi]],wd[,colvname])

} else {

newD<-newd0; ncg<-1;

f<-table(wd[,svname[xi]])

}

pred<-predict(mdl, data.frame(newD),se.fit=TRUE)

meany.pop0<-tapply(wd[,yvname[yi]],wd[,svname[xi]],function(z) mean(z,na.rm=TRUE))[1]

if (ylink[yi]=="logit") meany.pop0<-log(meany.pop0/(1-meany.pop0));

if (ylink[yi]=="log") meany.pop0<-log(meany.pop0);

shift<-meany.pop0-pred$fit[1]

y.fit <- pred$fit+shift

y.low <- pred$fit+shift-pred$se.fit*1.96

y.upp <- pred$fit+shift+pred$se.fit*1.96

y.pred<- cbind(y.fit,y.low,y.upp);

tmp.ylab<-paste("Mean of", yb[yi]);

cname.pred<-c("Mean","Mean.low","Mean.upp")

if (ylink[yi]=="logit") {

y.pred<-exp(y.pred); y.pred<-y.pred/(1+y.pred);

tmp.ylab<-paste("% of", yb[yi]);

cname.pred<-c("Rate","Rate.low","Rate.upp")

}

if (ylink[yi]=="log") {

y.pred<-exp(y.pred); tmp.ylab<-paste("% of", yb[yi]);

cname.pred<-c("Rate","Rate.low","Rate.upp")

}

y.fit<-y.pred[,1]; y.low<-y.pred[,2]; y.upp<-y.pred[,3]

if (!is.na(xvname[1])) {

tmp.ylab<-paste("Adjusted", tmp.ylab)

cname.pred<-paste("adj.", cname.pred, sep="")

}

y.pred<-round(y.pred,dec)

colnames(y.pred)<-cname.pred

y.pred <-cbind(newD[,svname[xi]],y.pred); colnames(y.pred)[1]<-svname[xi]

if (!is.na(colvname)) {

y.pred<-cbind(newD[,colvname],y.pred); colnames(y.pred)[1]<-colvname

}

y.pred<-rbind(colnames(y.pred),y.pred)

px<-c(20,1:9)

if (is.na(parm[1])) {tmp.col<-rainbow(ncg);tmp.col1<-c("red","blue");

} else {tmp.col<-rep("black",ncg);tmp.col1<-tmp.col}

pngf<-paste(ofname,yvar[yi],svname[xi],"adjmean.png",sep="_")

pdff<-paste(ofname,yvar[yi],svname[xi],"adjmean.pdf",sep="_")

if (ncg>1) {

xy<-legLocate(newD[,svname[xi]],c(y.fit))

png(pngf,width=720,height=560)

for (b in 1:ncg) {

x.tmp<-newD[newD[,colvname]==colv.lv[b],svname[xi]]

y.tmp<-y.fit[newD[,colvname]==colv.lv[b]]

if (b>1) par(new=TRUE)

plot(y.tmp~x.tmp,ylim=c(xy[3],xy[4]),xlim=c(xy[1],xy[2]),col=tmp.col[b],type="b", pch=px[b],

ylab=tmp.ylab, xlab=sb[xi])

}

legend(xy[5],xy[6],colv.lb,title=colvb,pch=px[1:ncg],bty="n",col=tmp.col)

dev.off()

pdf(pdff,width=pdfwd, height=pdfht, family="Helvetica");

for (b in 1:ncg) {

x.tmp<-newD[newD[,colvname]==colv.lv[b],svname[xi]]

y.tmp<-y.fit[newD[,colvname]==colv.lv[b]]

if (b>1) par(new=TRUE)

plot(y.tmp~x.tmp,ylim=c(xy[3],xy[4]),xlim=c(xy[1],xy[2]),col=tmp.col[b],type="b", pch=px[b],

ylab=tmp.ylab, xlab=sb[xi])

}

legend(xy[5],xy[6],colv.lb,title=colvb,pch=px[1:ncg],bty="n",col=tmp.col)

dev.off()

for (b in 1:ncg) {

x.tmp<-newD[newD[,colvname]==colv.lv[b],svname[xi]]

y.tmp<-y.fit[newD[,colvname]==colv.lv[b]]

y.lci<-y.low[newD[,colvname]==colv.lv[b]]

y.uci<-y.upp[newD[,colvname]==colv.lv[b]]

xy<-legLocate(c(x.tmp,x.tmp),c(y.lci,y.uci))

png(paste(ofname,yvar[yi],svname[xi],colvname,colv.lv[b],"CI.png",sep="_"),width=720,height=560)

plotCI(x.tmp,y=y.tmp,li=y.lci,ui=y.uci,pch=20,lwd=1,col=tmp.col[1], xlim=c(xy[1],xy[2]),ylim=c(xy[3],xy[4]),

ylab=tmp.ylab, xlab=sb[xi], main=paste(colvb, colv.lv[b],sep=": "))

lines(x.tmp,y.tmp,lty=2)

dev.off()

pdf(paste(ofname,yvar[yi],svname[xi],colvname,colv.lv[b],"CI.pdf",sep="_"),width=pdfwd, height=pdfht, family="Helvetica");

plotCI(x.tmp,y=y.tmp,li=y.lci,ui=y.uci,pch=20,lwd=1,col=tmp.col[1], xlim=c(xy[1],xy[2]),ylim=c(xy[3],xy[4]),

ylab=tmp.ylab, xlab=sb[xi], main=paste(colvb, colv.lv[b],sep=": "))

lines(x.tmp,y.tmp,lty=2)

dev.off()

}

} else {

x.tmp<-newD[,svname[xi]]

xy<-legLocate(c(x.tmp,x.tmp),c(y.low,y.upp))

png(pngf,width=720,height=560)

plotCI(x.tmp,y=y.fit,li=y.low,ui=y.upp,pch=20,lwd=1,col=tmp.col[1], xlim=c(xy[1],xy[2]),ylim=c(xy[3],xy[4]),

ylab=tmp.ylab, xlab=sb[xi], main="Adjusted mean & 95% CI")

lines(x.tmp,y.fit,lty=2)

dev.off()

pdf(pdff,width=pdfwd, height=pdfht, family="Helvetica");

plotCI(x.tmp,y=y.fit,li=y.low,ui=y.upp,pch=20,lwd=1,col=tmp.col[1], xlim=c(xy[1],xy[2]),ylim=c(xy[3],xy[4]),

ylab=tmp.ylab, xlab=sb[xi], main="Adjusted mean & 95% CI")

lines(x.tmp,y.fit,lty=2)

dev.off()

}

oo<-c("</br>Adjusted mean ",yb[yi], " by ", sb[xi], "<table border=3>",mat2htmltable(y.pred),"</table>")

gg<-c("<td>",yb[yi]," vs. ",sb[xi],"</br><a href=\"",pngf,"\" target=_BLANK><img src=\"",pngf,"\" width=320,height=320></a></td>")

return(list(oo,gg))

}

vlabelN<-(substr(vlabel,1,1)==" ");

vlabelZ<-vlabel[vlabelN];vlabelV<-vlabel[!vlabelN]

vnameV<-vname[!vlabelN];vnameZ<-vname[vlabelN]

ny<-length(yvname); yb<-vlabelV[match(yvname,vnameV)]; yb[is.na(yb)]<-yvname[is.na(yb)]

ns<-length(svname); sb<-vlabelV[match(svname,vnameV)]; sb[is.na(sb)]<-svname[is.na(sb)]

ssf<-rep(",fx=FALSE", ns); ssf[sdf>0]<-paste(",k=",sdf[sdf>0],sep="")

sxStr<-paste("s(",svname,ssf,sep="")

sxStr[slv>0]<-paste("factor(",svname[slv>0],")",sep="")

sxx<-paste("s(",svname,")",sep="")

sxx[slv>0]<-paste("factor(",svname[slv>0],")",sep="")

sxx<-matrix(sxx,ncol=1)

if (!is.na(colvname)) {

sxStr[slv==0]<-paste(sxStr[slv==0],",by=factor(", colvname, ")",sep="")

sxStr[slv>0]<-paste(sxStr[slv>0],"*factor(", colvname, ")",sep="")

colv.lv<-levels(factor(colv)); ncg<-length(colv.lv); colvb<-vlabel[vname==colvname];

colv.lb<-vlabelZ[match(paste(colvname,colv.lv,sep="."),vnameZ)]

colv.lb[is.na(colv.lb)]<-colv.lv[is.na(colv.lb)]

colvb<-vlabelV[match(colvname,vnameV)]; if (is.na(colvb)) colvb<-colvname;

sxplots<-NA; sxterms<-NA

for (i in (1:ns)) {

sxplots<-c(sxplots,paste(svar[i],"_",colvname,colv.lv,sep=""));

sxterms<-rbind(sxterms,paste(sxx[i,],":factor(",colvname,")",colv.lv,sep=""))

}

sxplots<-sxplots[-1]; sxterms<-matrix(sxterms[-1,],ncol=ncg)

sxterms<-cbind(paste("factor(",colvname,")",sep=""),sxterms)

} else {ncg<-1;sxplots<-svar; sxterms<-sxx;}

sxStr[slv==0]<-paste(sxStr[slv==0],")",sep="")

nx<-0

if (!is.na(xvname[1])) {

if (!is.na(colvname)) {xvname<-xvname[xvname!=colvname];}

nx<-length(xvname);

}

if (nx>0) {

xb<-vlabelV[match(xvname,vnameV)]; xb[is.na(xb)]<-xvname[is.na(xb)];

xvv<-xvname; xvv[xlv>2]<-paste("factor(",xvname[xlv>2],")",sep="")

if (!is.na(colvname)) {

xvv[sxf=="S" | sxf=="s"]<-paste("factor(",colvname,")*",xvv[sxf=="S" | sxf=="s"],sep="")

}

xv1<-paste(xvv,collapse="+")

}

if (is.na(par1)) par1<-1

if (ny!=ns & par1==2) par1<-1

if (par1==3) {nterms=ns*ncg*15+nx;} else {nterms=ncg*15+nx;}

w<-c("<html><head>","<meta http-equiv=\"Content-Type\" content=\"text/html\" charset=\"gb2312\" /></head><body>")

wtab<-"</br></br>Generalize additive models</br>"

wpng<-"</br><table>";

for (i in (1:ny)) {

if (par1!=3) {

wtmp<-"";

if (par1==2) {jstart<-i; jstop<-i;} else {jstart<-1; jstop<-ns;}

for (j in (jstart:jstop)) {

tmp.xx<-c(yvname[i],svname[j])

if (nx>0) tmp.xx<-c(tmp.xx,xvname)

if (!is.na(colvname[1])) tmp.xx<-c(tmp.xx,colvname)

wd<-WD[,tmp.xx];

wd<-wd[apply(is.na(wd),1,sum)==0,]

fml<-paste(yvname[i],"~",sxStr[j],sep="")

if (!is.na(colvname)) fml<-paste(fml,"+factor(",colvname,")",sep="")

if (nx>0) fml<-paste(fml,"+",xv1,sep="")

tmp.gam<-setgam(fml,i)

wtab<-c(wtab,paste("</br></br>Outcome:",yb[i]))

wtab<-c(wtab,paste("</br>Exposure:",sb[j]))

wtab<-c(wtab,gam2htmltable(tmp.gam))

if (slv[j]==0) {

wtmp<-c(wtmp,gam2pngs(tmp.gam,i,j))

} else {

stmp<-adjmean(tmp.gam,i,j)

wtmp<-c(wtmp,stmp[[2]])

wtab<-c(wtab,stmp[[1]])

}

}

wpng<-c(wpng,"<tr>",wtmp,"</tr>")

} else {

tmp.xx<-c(yvname[i],svname);

if (nx>0) tmp.xx<-c(tmp.xx,xvname)

if (!is.na(colvname[1])) tmp.xx<-c(tmp.xx,colvname)

wd<-WD[,tmp.xx];

wd<-wd[apply(is.na(wd),1,sum)==0,]

fml<-paste(yvname[i],"~",paste(sxStr,collapse="+"),sep="")

if (!is.na(colvname)) fml<-paste(fml,"+factor(",colvname,")",sep="")

if (nx>0) fml<-paste(fml,"+",xv1,sep="")

tmp.gam<-setgam(fml,i)

wtab<-c(wtab,paste("</br></br>Outcome:",yb[i]))

wtab<-c(wtab,gam2htmltable(tmp.gam))

if (sum(slv==0)>0) {

wpng<-c(wpng,"<tr>",gam2pngs(tmp.gam,i,0),"</tr>")

}

if (sum(slv>0)>0) {

for (k in 1:ns) {

if (slv[k]>0) {

stmp<-adjmean(tmp.gam,i,k)

wtab<-c(wtab,stmp[[1]])

wpng<-c(wpng,stmp[[2]])

}

}

}

}

}

wpng<-c(wpng,"</table>")

w<-c(w,wpng,wtab)

w<-c(w,"</body></html>")

fileConn<-file(paste(ofname,".htm",sep="")); writeLines(w, fileConn)
